# Simulating flexibility, variability and decentralisation with an integrated energy system model for Great Britain

**DOI:** 10.1038/s41598-023-31257-9

**Published:** 2023-03-23

**Authors:** Modassar Chaudry, Lahiru Jayasuriya, Jim W. Hall, Nick Jenkins, Nick Eyre, Sven Eggimann

**Affiliations:** 1 School of Engineering, Cardiff University, Queen’s Buildings, The Parade, Cardiff, Wales, CF24 3AA UK; 2 Environmental Change Institute, University of Oxford, South Parks Road, Oxford, OX1 3QY UK; 3 Urban Energy Systems Laboratory, Swiss Federal Laboratories for Materials Science and Technology, Empa, Dübendorf, Switzerland; 4 Department of Electrical and Electronics Technology, Faculty of Technology, Rajarata University of Sri Lanka, Mihintale, Sri Lanka

**Keywords:** Energy infrastructure, Energy grids and networks, Power distribution, Energy storage, Renewable energy

## Abstract

Energy system models allow the development and assessment of ambitious transition pathways towards a sustainable energy system. However, current models lack adequate spatial and temporal resolution to capture the implications of a shift to decentralised energy supply and storage across multiple local energy vectors to meet spatially variable energy demand. There is also a lack of representation of interactions with the transport sector as well as national and local energy system operation. Here, we bridge these gaps with a high-resolution system-of-systems modelling framework which is applied to Great Britain to simulate differences between the performance of decarbonised energy systems in 2050 through two distinct strategies, an electric strategy and a multi-vector strategy prioritising a mix of fuels, including hydrogen. Within these strategies, we simulated the impacts of decentralised operation of the energy system given the variability of wind and across flexibility options including demand side management, battery storage and vehicle to grid services. Decentralised operation was shown to improve operational flexibility and maximise utilisation of renewables, whose electricity supplies can be cost-effectively converted to hydrogen or stored in batteries to meet peak electricity demands, therefore reducing carbon-intensive generation and the requirement for investment in expanding the electricity transmission network capacity.

## Introduction

Meeting future energy demand whilst achieving the net-zero carbon emission target necessitates a power system that is decarbonised, whilst also nearly eliminating emissions from heating and cooling buildings, transportation and industry^1–3^. This requires a shift from the direct use of fossil fuels to the utilisation of low-carbon energy sources such as renewables (e.g. wind, solar, tidal and wave), nuclear, green/blue hydrogen, and bioenergy accompanied by efficiency improvements and reduction in overall energy consumption^4^. However, when switching to alternative fuels, various challenges persist such as the intermittency and variability of renewables supply resources, the limited availability of biofuels and the cost of hydrogen production^5,6^.

There has been considerable growth in local energy systems that utilise distributed energy supply resources such as wind and solar and rely on storage systems such as batteries to help meet local energy demand^7^. Distributed renewables can increasingly supply clean energy competitively at scale and have been identified as critical in meeting the net-zero emissions target^8,9^. Also, existing interdependencies between gas and electricity systems are evolving, for instance, the ongoing energy system decarbonisation in Great Britain (GB) anticipates a diminishing role for natural gas in electricity generation and heating^2^. On this basis, several studies^10–12^ have assessed the operation and planning of future low carbon integrated energy systems in GB. However, new interactions are emerging between local electricity, natural gas, district heat, and hydrogen supply systems through the deployment of new technologies (e.g. hydrogen boilers, fuel cells, electrolysers, hybrid heat pumps). In addition, consumer participation is increasing, for example by using rooftop solar PV and electric vehicle batteries to supply electricity back to the grid. Therefore, there is an urgency for integrated energy system analysis to represent and account for interactions between multiple energy supply systems and to explore alternative decentralised approaches to the operation of energy systems^13–15^.

Operating an electricity system with conventional large fossil-fuelled and nuclear power stations, with energy flowing in one direction from power stations through transmission and distribution lines to consumers, is typically referred to as centralised operation. This operational paradigm is likely to evolve with the increased use of distributed resources most of which are renewables (wind and PV) and storage systems in pursuit of a net-zero carbon energy system^2,16^. Traditional centralised system operations may not fully realise the benefits of distributed resources because they are connected within distribution and local energy systems. Therefore, decentralised operating approaches are being explored^17,18^. Gaining a better understanding of differences between centralised and decentralised operation under different energy strategies is critical to meeting the net-zero target. However, an integrated energy system representation, considering spatially distinct local energy systems and connections with national gas and electricity transmission systems models, is challenging^19^ due to the high spatial resolution inherent in modelling regional electricity, gas, heat and hydrogen supply systems and interdependencies with national systems^20^.

This study forms part of the ITRC-MISTRAL program^21^, which developed simulation capabilities^22^ to enable long-term cross-sectoral planning of sustainable and resilient infrastructure systems. Here, an integrated energy supply model based on the energy hub concept is described and showcased for the example of GB that considers interdependencies between multiple energy systems across national and local scales. Detailed spatio-temporal representations are used to explore energy storage systems (grid-connected systems, electric vehicle batteries, hydrogen storage) capturing the variability of renewable generation. The integration of energy demand and transport models through a simulation integration framework allows the representation of energy demand and energy-transport interactions such as the availability of EV (electric vehicle) batteries for vehicle-to-grid (V2G) services. This study demonstrates the importance of interactions between national and local energy systems to meet long-term net-zero carbon targets emphasising either a centralised or decentralised approach to energy dispatch. The interactions are assessed across two local energy supply strategies with distinctive future deployment of supply and demand-side technologies.

## Methodology

### Modelling of integrated energy supply systems

The modelling approach was demonstrated on the GB energy system. To simulate the operation of the GB energy system**,** the Combined Gas and Electricity Network (CGEN) model^23^ was used. CGEN models the natural gas and electricity transmission networks including their interaction through gas-fired power generators. Energy resource supplies, generation technologies, electricity and gas transmission networks, seasonal gas storage systems, variable generation of renewables and interconnectors are explicitly represented in the model. Energy supply at the transmission level meets demands from large industrial consumers and energy flows into distribution systems.

**Figure 1:**
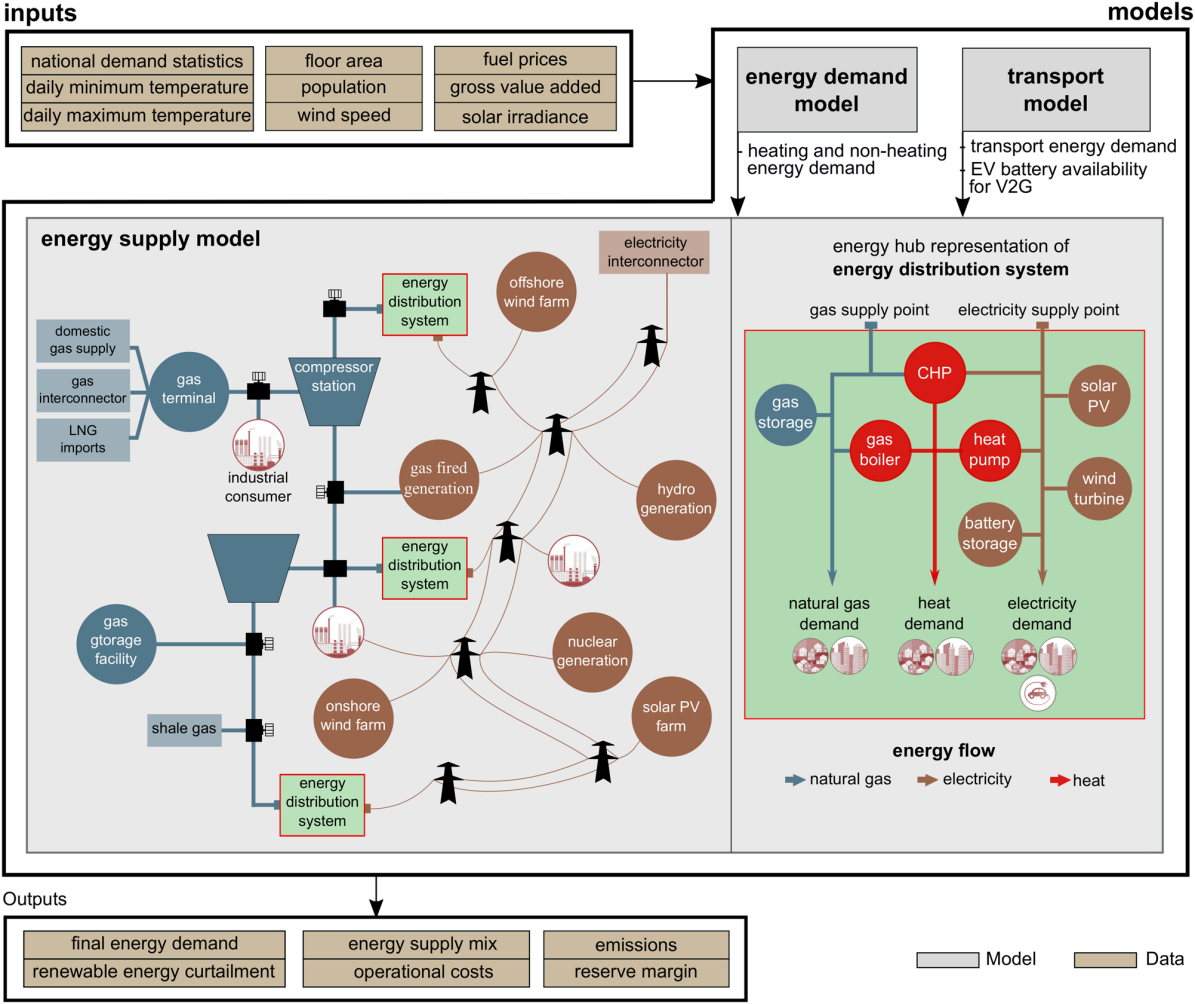
Integrated energy simulation framework linking an energy supply, energy demand, and a transport model. A stylised representation of the energy supply system model is shown, including the energy hub representation of local energy distribution systems.

The CGEN model was upgraded^24^ to include the representation of spatially distinct electricity, natural gas, heat and hydrogen distribution systems by adopting the “energy-hub”^25^ concept (see Fig. 1). An energy hub provides an aggregated view of local electricity, gas, heat and hydrogen distribution supply systems and energy demand within its regional geographic boundary. The energy hubs are connected with gas and electricity transmission networks through grid supply points within a region. Energy hubs utilise distributed energy resources which includes both end-user level technologies such as rooftop PV, heat pumps and network-connected technologies such as wind farms, large CHP units, storage and transmission grid supplies to meet residential, commercial, and transport energy demand. Constraints of each technology and network energy flow capacities are modelled.

The integrated energy supply system (transmission and energy hubs) model minimises total operational costs to meet energy demand as given in Eq. (1) and is applied for the centralised energy system operation. The cost minimisation is subjected to constraints derived from the operational characteristics of assets (e.g. power stations, transmission lines) in both national (transmission) and energy hub systems (distribution) while ensuring the balance between energy supply and demand. The operational costs at each time step *t*, are derived from the natural gas ${C}_{t}^{Gas\_Tran}$ and electricity ${C}_{t}^{Elec\_Tran}$ transmission networks, energy hubs ${C}_{t}^{Energy{Hub}_{k}}$, carbon costs ${C}_{t}^{Carbon}$ and unserved energy (${C}_{t}^{unserved\_energy}$) over the simulation time horizon. All the individual operational cost components are expanded in subsequent Eqs. (2) to (4). The time step $t$, represents an hour and the simulation time horizon covers a representative week for each season of a year.

1 $$Objective=min\sum_{t}\left\{{C}_{t}^{Elec\_Tran}+ {C}_{t}^{Gas\_Tran}+\sum {C}_{t}^{Energy{Hub}_{k}}+{C}_{t}^{Carbon}+{C}_{t}^{unserved\_energy}\right\}$$

where ${C}_{t}^{Elec\_Tran}$ as modelled in Eq. (2) includes power generation costs ${C}_{j}^{gen}$ such as fuel costs, operation and maintenance costs of power generator $j$ (excluding interconnectors) for generating power ${P}_{j,t}$, costs of importing power ${P}_{i,t}^{imp}$ for a unit price ${C}_{i}^{imp}$ and the revenues from exporting power ${P}_{i,t}^{exp}$ for a unit price ${C}_{i}^{exp}$ via an interconnector link $i$.

2 $${C}_{t}^{Elec\_Tran}=\sum_{j}{C}_{j}^{gen}{P}_{j,t}+ \sum_{i}\left({C}_{i}^{imp} {P}_{i,t}^{imp}-{{C}_{i}^{exp}P}_{i,t}^{exp}\right)$$

Equation (3) represents ${C}_{t}^{Gas\_Tran}$, this includes the cost of gas supply from terminal $a$ at time $t$ calculated by the gas price ${C}_{a,t}^{gas}$ and volume of gas supplied ${Q}_{a,t}^{sup}$, the cost of operating a gas storage facility $u$ calculated by the gas volume injected ${Q}_{u,t}^{I}$ or withdrawn ${Q}_{u,t}^{W}$ at time t and the cost of gas injection ${C}_{u}^{I}$ or withdrawal ${C}_{u}^{W}$.

3 $${C}_{t}^{Gas\_Tran}=\sum_{a}{C}_{a,t}^{gas}{Q}_{a,t}^{sup}+\sum_{u}\left\{{C}_{u}^{W}{Q}_{u,t}^{W}+{C}_{u}^{I}{Q}_{u,t}^{I}\right\}$$

The energy hub costs $\left({C}_{t}^{Energy{Hub}_{i}}\right)$ of operating integrated electricity, natural gas, heat and hydrogen distribution systems are modelled in Eq. (4), which includes operational costs of distributed technologies including fixed and variable costs $\left({C}_{i}^{f\&v}\right)$ of technology ($i$) with respect to energy outputs $\left({E}_{i, output, t}\right)$, fuel costs for biomass $\left({C}_{bio}^{fuel}\right)$ and solid waste $\left({C}_{w}^{fuel}\right)$.

4 $${C}_{t}^{Energy{Hub}_{k}}=\left\{\sum_{i}^{\left\{Tech\right\}}{E}_{i, output, t}\times {C}_{i}^{f\&v}\right\} + \left\{\sum_{j}^{\left\{bio, w\right\}}{E}_{j,t}\times {C}_{j}^{fuel}\right\}$$

Carbon costs ${C}_{t}^{Carbon}$ were applied across electricity generation, heat supply, hydrogen production and non-heating end-uses of fuels (natural gas, oil, solid fuel). Within both national and local energy systems, penalty costs were applied for unserved energy ${C}_{t}^{unserved\_energy}$ demand.

The optimisation problem of the energy supply model (Eq. 1) was developed and solved using the commercial optimisation tool Fico Xpress^26^. The Xpress Sequential Linear Programming (SLP) solver for non-linear programming was used to minimise the objective function over the entire simulation time horizon. The inbuilt Xpress SLP solver has been used for different complex non-linear optimisation problems^10–12^ based on the CGEN model. Since the energy supply model used is an extension of the CGEN model, the same optimisation tool and solver was used. The efficacy of other solvers was not considered within the scope of this study.

Variable output from renewable energy supplies and curtailments are modelled using time series of hourly wind speed and solar irradiance. Spatial and temporal variability of wind speed and solar irradiance are accounted for in the GB electricity transmission network and local energy hubs. For this, the “Weather@Home”^29^ data set was used to obtain forward projections of wind speed, solar irradiance and temperature. The data for these parameters are available in a daily time granularity for a historic baseline (1900–2006) and future years (2020–2050) across numerous future climate change scenarios (~ 100 realisations). The historic baseline data has been validated^30,31^ and future projections are in line with UK Climate Projections 2009^32^. A 10 km × 10 km grid is available across GB providing weather parameters at each grid point. From this, the data from grid points closest to the electricity bus bars, and the grid points at the centre of each energy hub region were chosen. The daily weather data was down-scaled to hourly data using historical hourly weather patterns from the Met Office data archive^33^.

Given the wind speed ${v}_{t}$ , Eq. (5) calculates the power output ${P}_{k,t}$ from a wind turbine $k$ with a rated capacity ${P}_{k}^{rated}$ as follows. Cut-in $\left({v}^{cut-in}\right)$, cut-off $\left({v}^{cut-off}\right)$ and rated $\left({v}^{rated}\right)$ wind speeds were chosen from product catalogues of wind turbine manufacturers^34,35^. The provided equation reflects a linear approximation of wind speed and power output, which in reality follows an exponential relationship^36^. A quantification of the approximation errors is provided in Supplementary note E.

5 $${P}_{k,t}=\left\{\begin{array}{c}0 ; \,if\,{v}_{t}\le {v}^{cut-in} \,or\, {v}_{t}\ge {v}^{cut-off}\\ \\ \left(\frac{{v}_{t}-{v}^{cut-in}}{{v}^{rated}-{v}^{cut-in}}\right){P}_{k}^{rated} ;\,if\, {v}^{cut-in}\le {v}_{t}\le {v}^{rated}\\ \\ {P}_{k}^{rated} ; \,if\, {v}^{rated}\le {v}_{t}\le {v}^{cut-off}\end{array}\right.$$

The power output from a solar PV array was modelled using Eq. (6), given hourly solar irradiance $({I}_{t})$ as inputs.

6 $${P}_{k,t}={A}_{k}\times {\eta }_{k}\times P{R}_{k} \times {I}_{t}$$

Here, ${\eta }_{k}$ is the efficiency of the solar PV array $k$ and $P{R}_{k}$ is the performance ratio of the array which considers additional losses, e.g., losses due to high cell temperature. An average value of 0.7 for $P{R}_{k}$ and 0.2 for ${\eta }_{k}$ were used^37^. In reality $P{R}_{k}$ should be a dynamic coefficient as it varies according to several factors including the ambient temperature and the level of irradiance received. To reduce the modelling complexity, $P{R}_{k}$ was kept as a static coefficient by using an average value. The area of the PV array is described by assuming the array is composed of standard 200 W solar PV panels. Considering the area of a 200 W panel (the typical area is 1.24 m^2^), Eq. (7) calculates the total area of a PV array,

7 $${A}_{k}={P}_{k}^{rated}\left(kW\right)\times \frac{1.24 }{0.2}(\frac{{m}^{2}}{kW})$$

### Energy-Transport system interactions

A simulation modelling integration framework (SMIF)^38^ is used to link the energy supply model to energy demand^39^ and transport^40^ models. These two models collectively provide inputs such as heat and building energy demand, along with transport energy demand, which are met by energy supplies as determined by the energy supply model. SMIF integrates data inputs and outputs between the various models and accounts for the differences in spatial and temporal resolution. A stylised representation of the energy transmission and distribution systems, energy demand and transport model interactions are illustrated in Fig. 1.

The model set-up offers a highly disaggregated temporal (hourly) and spatial (Local Authority District level) representation of the energy system. This allows detailed analysis of a future energy system under various strategies such as integration of large capacity of renewables, expansion of community and distributed generation, and greater consumer participation through demand side management (DSM) including smart charging and vehicle-to-grid electricity supply from electric vehicle batteries.

### Centralised and decentralised operation of local and national energy systems

**Figure 2:**
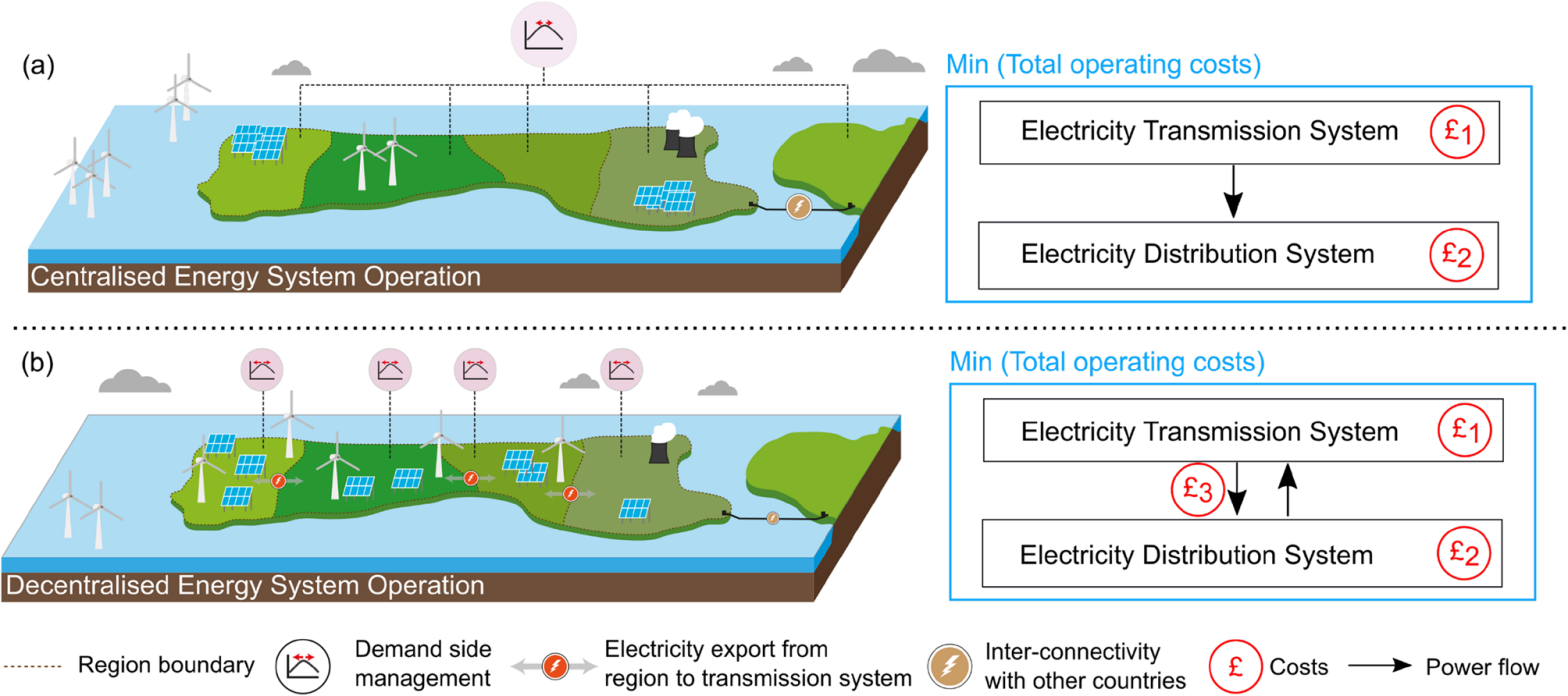
Stylised representation of (**a**) centralised and (**b**) decentralised energy system operation and illustration of differences in the cost optimisation modelling approach. The level of prominence given to energy technologies such as large power stations, distributed generation, demand-side management, and interconnectivity is illustrated.

The characteristics of an energy system with centralised or decentralised operation are illustrated in Fig. 2. In centralised operation (Fig. 2a), the system operator can use a mix of transmission and distribution grid-connected supply sources to meet the energy demands such that the total operating costs are minimised (see Eq. 1). The cost minimisation is subject to system operational characteristics and constraints such as generation plant and network capacities. Additionally, when simulating a centralised energy system, it is assumed that energy can only flow from transmission to the distribution system. Centralised system operation mainly utilises large capacity power stations and increased interconnection with other countries while less prominence is given to distributed generation and storage, and DSM.

In decentralised operation (Fig. 2b), two-way energy flows from electricity transmission and distribution systems and vice-versa are permitted. The objective function for decentralised operation is represented by Eq. (8) which penalises electricity flows from transmission to the distribution system using a fixed tariff $\left({C}_{t}^{Tran-EH}\right)$ and thus encourages distributed energy resources, technologies, and storage systems to perform a more prominent role in the overall energy system while decreasing the use of large power stations and interconnectors.

The energy flows from distribution to the transmission system are constrained by the capacity of grid supply points. Additionally, DSM is given prominence and grants the system operator more control of distributed resources.

8 $$Objectiv{e}_{Decentralised}=min\sum_{t}\left\{{C}_{t}^{Elec \_Tran}+ {C}_{t}^{Gas \_Tran}+\sum {C}_{t}^{Energy{Hub}_{k}}+{C}_{t}^{Carbon}+{C}_{t}^{unserved\_energy}+{C}_{t}^{Tran-EH}\right\}$$

The approach of penalising energy flows maximises the local energy generation and minimises system operating costs whilst modifications to the existing optimisation problem and additional constraints are kept to a minimum. The addition of penalty costs also reduces complexity and therefore computational time of the existing optimisation problem when simulating in decentralised operational mode.

### Development of national and local energy system strategies for net-zero emissions

**Figure 3:**
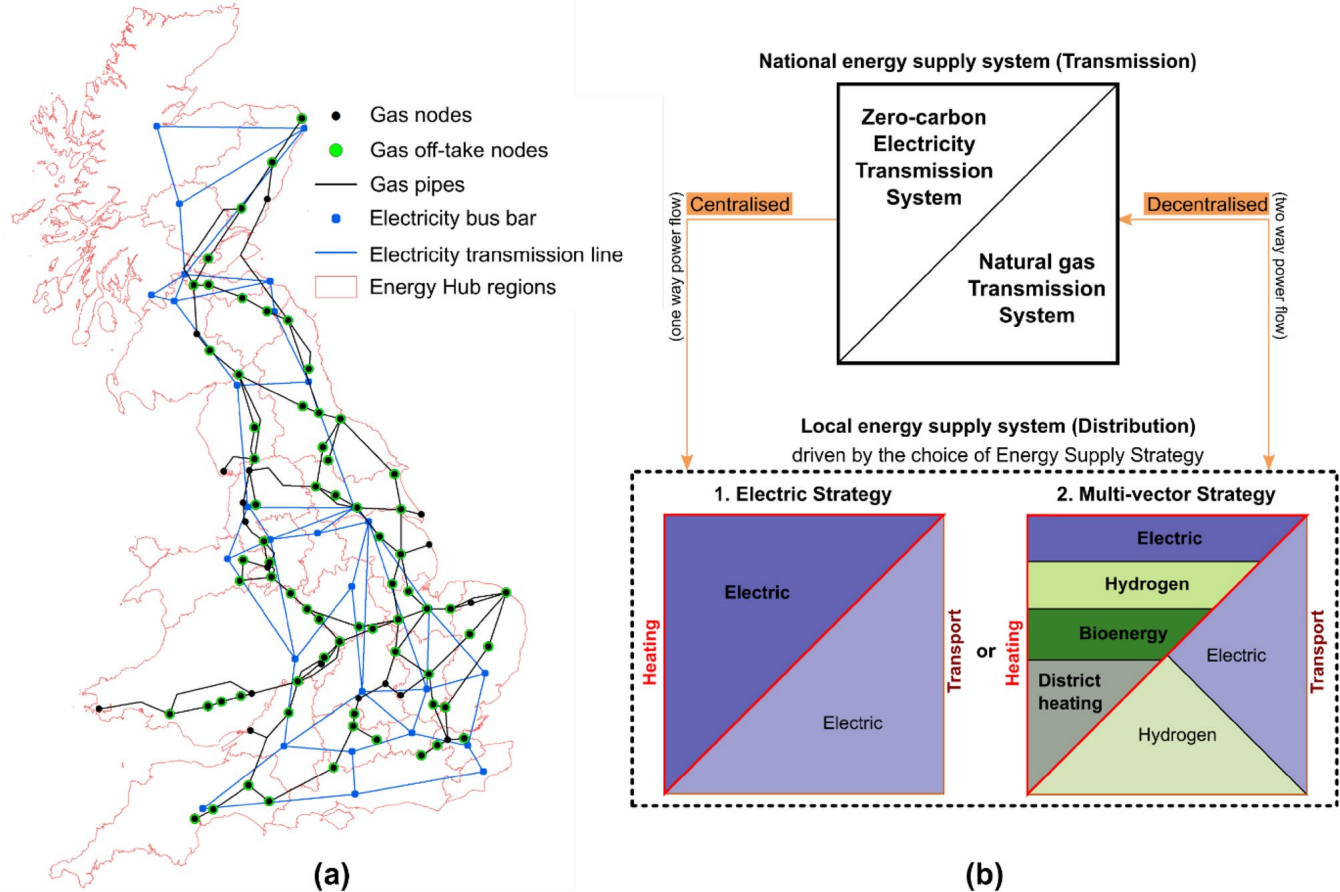
(**a**) Electricity and natural gas transmission network representation, and energy hub geographical regions representing local energy systems of Great Britain (**b**) Outline of energy supply strategies in 2050 defined through a combination of heat and transport decarbonisation options for centralised and decentralised operation.

The GB energy system was used to demonstrate interactions between national and local energy systems for two future strategies. The spatial representation of the electricity and natural gas transmission network and energy hub geographic regions is shown in Fig. 3a. The national gas and electricity transmission systems were configured such that the technology and network capacities meet the net zero emissions target in 2050. The energy supply capacity data were taken from studies by the system operator^41^ and the UK Climate Change Committee^15^. Figure 3b outlines the energy supply strategies used in this study.

Given the uncertainties surrounding the decarbonisation of heating and transport, two energy supply strategies were defined. The two strategies span the likely scenario space, i.e., from large amounts of electrification to the use of multiple-energy vectors to meet the heating and transportation energy demand. In the *Electric Strategy,* both heating and transportation are electrified. The transportation sector is dominated by battery electric vehicles while heating is done with heat pumps, hybrid heat pumps, electric boilers, and resistive heaters. The *Multi-vector Strategy* utilises combined heat and power (CHP) units connected to district heating networks that are driven by biomass, waste, and hydrogen. Additionally, hydrogen boilers and heat pumps are used to decarbonise heat while a mix of hydrogen fuel cells and battery electric vehicles are used to decarbonise transportation. A summary of the local energy supply strategies is outlined in Table 1 which considers technology uptakes, maturity, annual build rates, annual and peak heat demand and capacity margin factors^15,41–43^.

**Table 1:** Summary of assumptions for the Electric and Multi-vector strategies.

Sector	(1). Electric Strategy	(2). Multi-vector Strategy
Heating	Heat is supplied completely by electricity using heat pumps, resistive heating, electric boilers, and hybrid heat pumps (i.e. combined electric heat pump and a gas boiler).	Heat is supplied by utilising several energy vectors and technologies. Building heat supply is either from hydrogen boilers or air source heat pumps or homes are connected to a district heating network where the majority is run from hydrogen fuel cells, biomass CHP and waste CHP units. Only few natural gas CHP run district heating networks exist.
Electricity generation	National electricity system is predominantly operated by generation from renewables (wind and PV) and nuclear plants. Interconnectors, natural gas and biomass plants equipped with CCS provides support for variations in demand and renewable supply. Distributed generation within the Energy Hubs is mainly from wind, solar photovoltaic (PV) with access to grid-scale battery storage systems.	National electricity system is predominantly operated by generation from renewables (wind and PV) and nuclear plants. Interconnectors, natural gas and biomass plants equipped with CCS provides support for variations in demand and renewable supply. Distributed generation is a combination of renewables equipped with batteries and CHP units in district heating applications (heat demand-driven CHP operation is assumed).
Natural gas supply	Transmission grid supplies are available with limited gas storage facilities.	Transmission grid supplies are available with sufficient large gas storage facilities considering the scale of hydrogen production using large SMR facilities. Most of the natural gas distribution networks around the country are re-purposed to transport hydrogen and some remain to provide natural gas for gas CHPs.
Hydrogen	Small scale hydrogen production and storage facilities are installed mainly around the demand centres. Hydrogen is supplied via new hydrogen pipelines and re-purposed gas distribution pipes.	A large capacity of electrolysers and SMR with CCS facilities are installed to produce hydrogen. Hydrogen production from SMR and electrolysers have access to hydrogen storage facilities. Hydrogen is supplied via new hydrogen pipelines and re-purposed gas distribution pipes.
Transport	All cars and vans are electrified. Majority of the HGVs are plug-in hybrid electric vehicles, where the remaining run-on fuel-cells. A small portion of the HGV fleet will keep using internal combustion engines.	About half of all cars run on electricity where the remaining will be a mix of plug-in hybrids, and hydrogen fuel-cell vehicles. Just over half of all vans will be electric, and the remaining will use hydrogen fuel-cells. Half of the HGV fleet will convert to run on hydrogen fuel-cells and reminder will be plug-in hybrids.

The energy supply capacities for each strategy were adopted from studies performed by the GB electricity system operator^41^ and the UK Climate Change Committee ^15^ (see Supplementary Note B) such that the net-zero emission target is met. Our simulations focus on the operation of the energy system with given capacities and do not include investment costs. Most energy system planning studies explore how the present system would evolve and calculate optimal capacities considering capital costs to meet the net-zero target. Our focus is on the operational performance of different future energy systems in 2050 whilst meeting the net-zero target, in particular illustrating how flexibility, variability and decentralisation impacts system operation, costs, and emissions.

Simulations were performed for GB with each energy supply strategy applied to all energy hubs simultaneously covering centralised and decentralised operation for the simulation year 2050. We chose a historic year (2015) to enable the calibration of models with known data. The simulation of each year considered four seasons with hourly time resolution. Relevant assumptions and input data are given in the Supplementary Notes.

## Results and discussion

### Spatio-temporal energy system impacts of Electric and Multi-vector strategies

**Figure 4:**
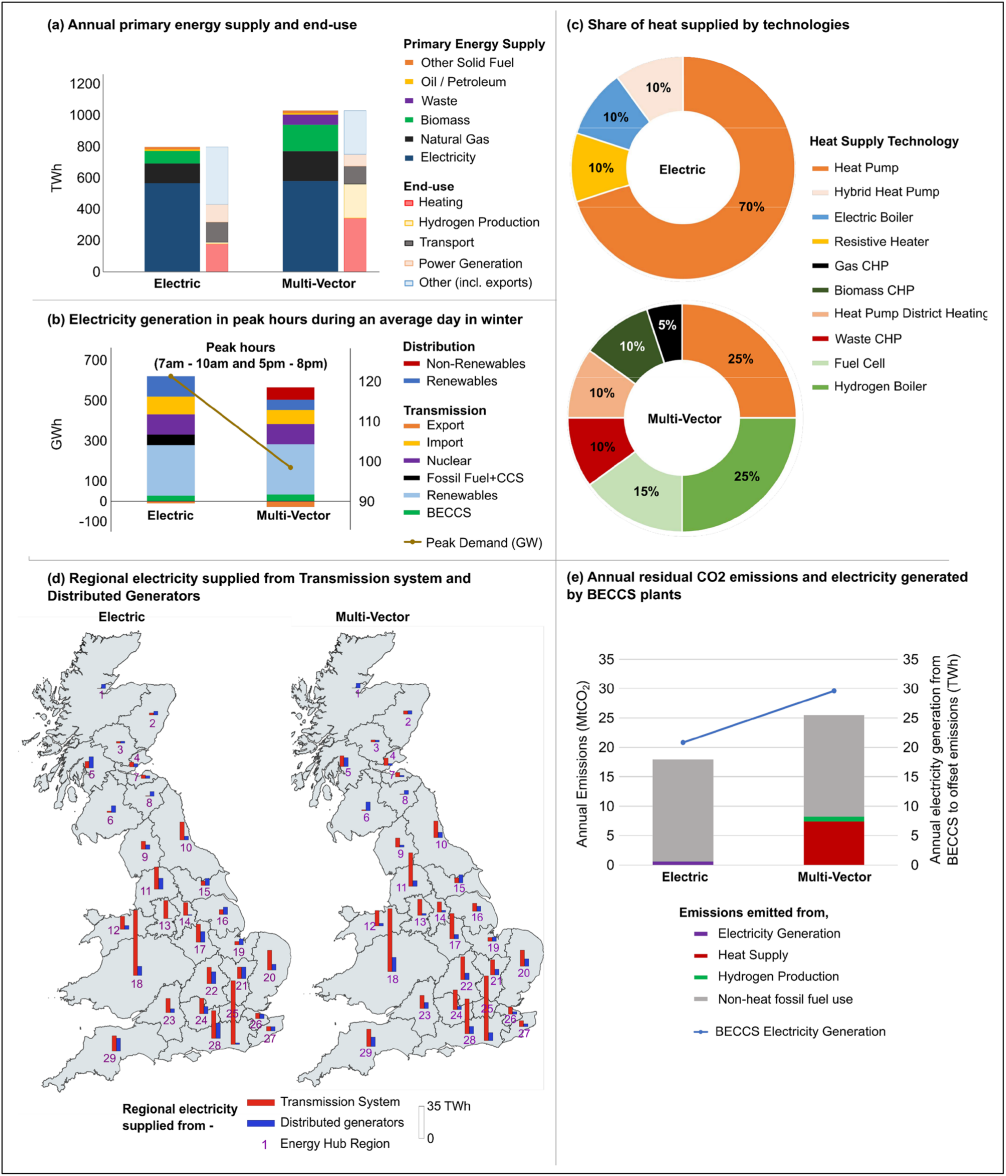
Comparison of different future energy supply strategies (Multi-vector and Electric) assuming centralised system operation. (**a**) Annual primary energy supply and consumption by end-use, (**b**) electricity generation in peak hours during a typical day in winter, (**c**) share of heat supplied by technology, (**d**) regional electricity supply split between the transmission system and distributed generators to meet local electricity demands (**e**) annual residual CO_2_ emissions, and electricity generated from Bioenergy with Carbon Capture and Storage (BECCS) plant for the Energy Supply Strategies in the year 2050.

The Electric strategy results in lower primary energy demand (Fig. 4a) compared to 2015 (45% reduction) and the Multi-vector Strategy in 2050, facilitated through utilisation of heat pumps and efficiency gains.

Hydrogen production through renewable electricity and natural gas increases the requirement for primary energy supplies in the Multi-vector Strategy. Additionally, compared with electric vehicles hydrogen fuel-cell vehicles are less efficient and require greater energy supplies to meet transport energy demand.

Compared to the base year 2015, electrification of heating and transport in the Electric Strategy results in a doubling of annual and peak electricity demand to ~ 600TWh and 120GW in 2050 (Fig. 4b). In the Multi-vector Strategy, annual and peak electricity demand in 2050 is lower as a mixture of hydrogen and biomass fuels are used to meet heating and transport demands. The strategies demonstrate two distinct pathways in which heat demand can be met using a mix of different fuels and technologies (Fig. 4c).

Generation from transmission connected onshore and offshore wind farms is 34TWh greater in the Multi-vector Strategy compared with the Electric Strategy with considerably lower curtailments. This is mainly due to the additional options for electricity consumption in the Multi-vector Strategy such as the production of hydrogen through electrolysis. For both strategies, interconnector flows become more variable and more bi-directional flows occur throughout a typical winter day in 2050. During off-peak periods, interconnectors mainly export electricity, whereas during peak periods electricity is imported to balance the system alongside ramping of CCGT plants equipped with carbon capture and storage.

By 2050 the capacity of generation plants connected to the distribution system grows by 75GW from the base year for both strategies. Annual distributed electricity generation increases on average seven-fold from base year to 2050 and meets ~ 30% of total electricity demand. The use of CHP units is greatest in the Multi-vector Strategy generating 40TWh annually. In the Electric Strategy, distributed renewable generation is facilitated through the utilisation of batteries.

The model captured the spatial variability for electricity generation as shown in Fig. 4d. Each region is split between transmission flows and distributed generation originating within the region to meet local electricity demands. Given lower overall local electricity demands in northern GB (regions 1–8), large electricity flows are observed to southern GB regions from electricity generated in the north by offshore and onshore wind and hydro-electric plants. The spatial explicit energy supply representation assists the identification of potential bottlenecks in transmission capacity and allows the system operator to utilise the available generation to prevent load shedding in regions with lower levels of distributed generation. The analysis reveals regions with high hydrogen demand (e.g., region 18) show large consumption of electricity for hydrogen production through electrolysis in the Multi-vector Strategy. Regions with large CHP capacity show increased use of distributed generation as well as in regions with large capacity of PV and onshore wind with access to batteries (e.g., regions 28 and 29).

CO_2_ emissions in 2050 are net zero in both energy supply strategies. Figure 4e shows the residual annual emissions which result from the remaining direct use of fossil fuels as well as what is left from the CCS process (assuming 90% efficiency in CO_2_ capture) across electricity generation, hydrogen production, heat supply and industrial sector before being offset by negative emissions from electricity generated from BECCS plants. Residual emissions determine the required capacity of BECCS plants and biomass resources. For instance, the greater the residual emissions, the greater the requirement for BECCS capacity and biomass which impacts overall system costs. In 2050, residual CO_2_ emissions are larger in the Multi-vector Strategy but are reduced by more than 85% compared with 2015.

The Multi-vector Strategy maximises the use of available renewable resources, particularly with the production of hydrogen during off-peak hours and the use of hydrogen storage facilities. However, the use of natural gas to produce hydrogen in large quantities for heating and transport results in additional residual CO_2_ emissions leading to greater use of BECCS plants and consequently operational costs. These costs nullify some of the cost savings realised through co-generating heat and electricity within the Energy Hubs.

### Decentralised operation of energy systems

**Figure 5:**
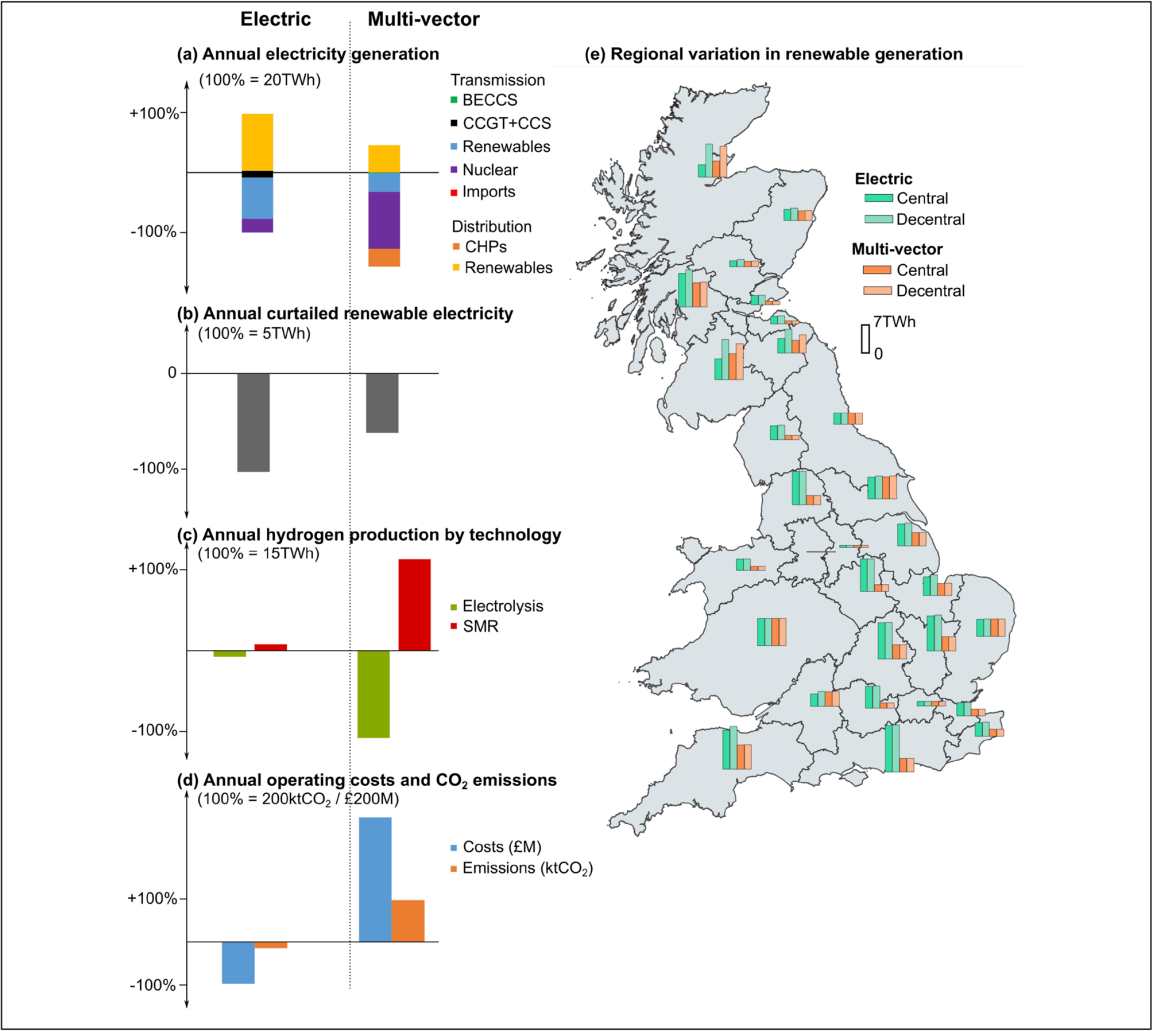
Figures (**a**–**d**) show the percentage change of a switch from centralised to decentralised energy system operation for: (**a**) Electricity generation from transmission and distributed generators, (**b**) Renewables generation curtailed (wind and PV), (**c**) Hydrogen production by technology, (**d**) Operational costs and residual CO_2_ emissions, and (**e**) Regional variations in renewable generation for centralised and decentralised operation in 2050.

Decentralised operation in both the Electric and Multi-vector Strategy maximises the use of regional renewables. Therefore, switching from centralised to decentralised system operation leads to a reduction in annual electricity generation from plants connected to the transmission system and a decrease in electrical power flows from transmission to distribution systems (Fig. 5a).

Both strategies reduce annual renewable electricity curtailments (Fig. 5b). Overall, renewable electricity curtailments are lower in the Multi-vector Strategy due to the production of hydrogen via electrolysers. The increase in renewables connected to the distribution system in the Electric Strategy compensates for the lower use of renewables connected to the transmission system. Reduction in curtailments is greatest in the Electric Strategy as grid-scale distributed battery storage systems manage the temporal mismatch of distributed renewable generation and local demand. This shows that the installation of sufficient battery storage capacity allows optimal utilisation of renewable energy.

In the Multi-vector Strategy, electricity from renewables connected to the distribution system meets the bulk of local electricity demands except for hydrogen production. Electricity imported into Energy Hubs is mainly used to produce hydrogen, although compared with centralised operation, hydrogen production through electrolysis is reduced. This is mainly due to the costs attached to electricity flows from the transmission system. Conversely, Steam Methane Reformation (SMR) facilities connected to the gas transmission system are increasingly utilised to produce hydrogen as shown in Fig. 5c. In decentralised operation, the competitiveness between costs of electricity flows from the transmission system to Energy Hubs and hydrogen production technologies is critical for optimal selection of hydrogen production methods.

Decentralised operation also showed an impact on an energy system with large hydrogen demands. When renewables meet the heating and non-heating electricity demands, hydrogen production is dominated by SMR facilities as there is limited surplus electricity available from renewables to produce hydrogen through electrolysis. This shift in the production of hydrogen increases the requirement for natural gas supplies, and consequently, overall system operating costs in 2050 rise by ~ £580 million and residual emissions by ~ 200 ktCO_2_ (Fig. 5d). In contrast, decentralised operation of the Electric Strategy led to a reduction in operating costs of ~ £200 million and residual emissions by ~ 30 ktCO_2_.

The regional modelling showed spatial variation in the utilisation of distributed renewable electricity (Fig. 5e). Particularly in northern GB, which has large installation of renewables, decentralised operation was able to maximise its generation. However, regions with limited renewable generation capacity, large demand centres and low distribution system capacity show only minor differences between centralised and decentralised operation.

### Impact of wind variability on the operation of an energy system

The operation of future energy systems is faced with inherent uncertainties in supply and demand. Assessing uncertainties of renewable energy generation, particularly wind, is critical as they are anticipated to increase due to climate change targets^44^.

To explore the impact of wind variability, a ± 20% change of hourly wind speeds was examined. The extent to which centralised and decentralised operation encourages integrated operation across different vectors (electricity, natural gas, hydrogen) and the use of storage systems during high and low levels of wind generation was investigated for the energy strategies over a typical winter day in 2050.

**Figure 6:**
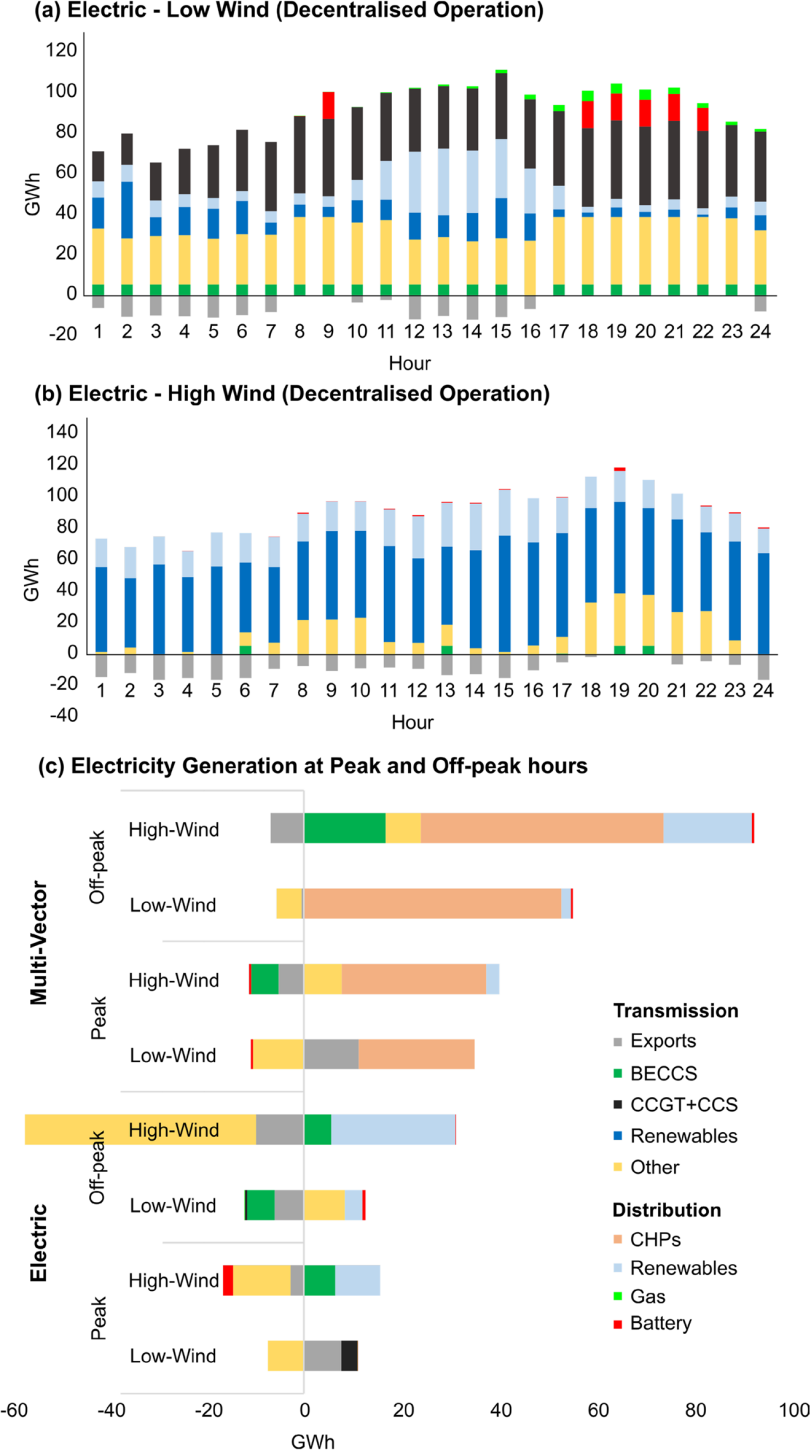
Impact of (**a**) low-wind, and (**b**) high-wind on the hourly electricity generation during a day in winter in the Electric Strategy with decentralised operation. (**c**) Impact of switching from centralised to decentralised system operation on electricity generated at peak (morning 06.00–09.00 and evening 17.00 to 20.00) and off-peak hours during a day in winter under low/high wind across the two strategies in 2050.

When both heating and transport is electrified, a significant shortfall in wind increases the requirement for fossil-fuelled plants such as CCGT + CCS and dedicated distributed gas-fired generators as battery storage systems, nuclear and interconnector electricity supplies are supplying energy at maximum capacity (Fig. 6a). This requires additional natural gas, gas line-pack and short-term storage which increases operational costs. No significant benefit was observed in operating the energy system in decentralised operational mode during times of low-wind generation regardless of the energy supply strategy. The upward ramping of CHPs operating on biomass and waste in the Multi-vector Strategy showed the ability to mitigate the impacts of low wind generation without utilising CCGT + CCS or other fossil-fuelled distributed plants. When there is abundant renewable generation available (Fig. 6b), it meets more than 70% of the electricity demand and as a result, most other generation plants operate at lower capacity factors.

In contrast, a decentralised energy system was able to maximise the utilisation of wind generation when it was plentiful regardless of the strategy (Fig. 6c). For this, the Electric Strategy utilises distributed battery storage systems to meet local electricity demands for heating and transport. In a Multi-vector Strategy, additional wind generation is used to increase hydrogen production through electrolysis and reduces the use of steam methane reformation and consequently natural gas supply. Integrated operation of electricity and hydrogen systems utilises hydrogen produced from renewable electricity during off-peak hours to be stored and used when demand increases. Decentralised operation improves the system’s ability to maximise the utilisation of renewables at the expense of higher-marginal cost generation plants and therefore reduce operating costs. Decentralised operation with plentiful wind generation was shown to lower operating costs by £275 million for the Electric Strategy, and £50 million for the Multi-vector Strategy in 2050.

The advantages of decentralised operation are illustrated in the case of plentiful wind generation either through the utilisation of distributed battery storage systems for meeting local heating and transport demands or an increase in hydrogen production through electrolysis which reduces the use of natural gas.

### Operational flexibility of an integrated energy system

Increased electrification of heating and transport amplifies the dependency on electricity generation and therefore the requirement for flexibility options to operate reliably subject to uncertainties in energy supply^45^. Options to improve flexibility of the energy system include smart charging and vehicle to grid electricity supply from electric vehicles, DSM schemes and additional battery storage capacity which allows the system operator to adjust supply and consumption to balance the energy system.

**Figure 7:**
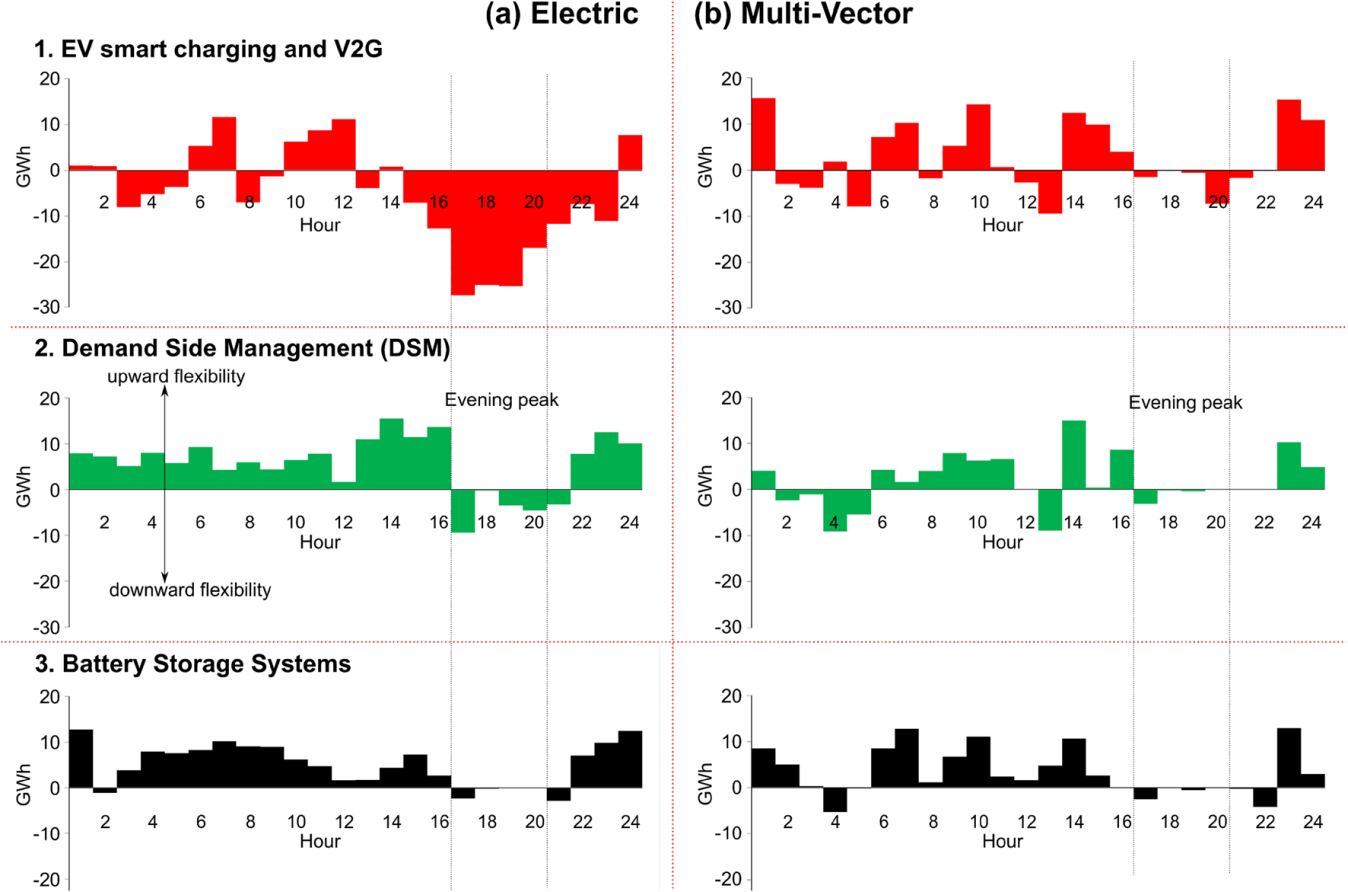
The change in hourly electricity flows from the transmission to local energy systems over a typical winter day with the use of different flexibility options across the two strategies in 2050: (1) electric vehicle smart charging with V2G, (2) DSM, and (3) large grid-scale batteries. Positive values convey an increase from transmission to distribution electricity flows due to the flexibility option during decentralised operation, and negative values indicate that electricity flows have decreased.

The ability to change the net electricity flows from transmission to local energy systems, conveys the extent to which flexibility can be provided locally to the whole energy system. Decentralised system operation with different flexibility options was modelled across Electric (Fig. 7a) and Multi-vector (Fig. 7b) strategies during a typical day in winter assuming low levels of renewables generation.

Flexibility is largely provided from local energy systems through smart charging of electric vehicle and V2G services in the Electric Strategy during the evening peak (17.00–20.00). This is due to electric vehicles shifting their charging demand to periods when there is excess generation from renewables. The flexibility provided by DSM during the same period is lower due to the smaller magnitude of non-heating electricity demands (washing machines and dishwashers etc.) that can be shifted compared to electric vehicles. There was no significant advantage in operating additional battery storage capacity with low levels of overall renewable generation.

The Multi-vector Strategy did not show significant use of flexibility services from local energy systems during peak hours as electricity demand is lower when compared to the Electric Strategy. The Multi-vector Strategy provides flexibility by using excess renewable electricity stored in the form of hydrogen within hydrogen storage facilities.

The availability of flexibility from local energy systems whilst interacting with the national electricity transmission system was found to reduce the use of fossil-fuelled peaking plants (e.g., CCGT + CCS) and further diminish renewable electricity curtailments. Flexibility from smart charging of electric vehicles and V2G resulted in the largest annual cost savings of £304 million in the Electric Strategy in 2050.

Decentralised operation was shown to offer options such as smart charging, V2G services and DSM, therefore additional flexibility from local energy systems. This resulted in a reduction of fossil-fuel power generation and therefore associated emissions, energy curtailments and operational costs.

## Conclusions

A modelling framework was introduced and applied to GB that integrates an energy demand and transport model with an energy supply system model. Whereas the integrated framework was developed for GB, and even though we showcased its capabilities for this particular case study context, the conceptual framework could be transferred to other countries. This would require the availability and integration of a country-specific energy supply, energy demand and transportation model. Whereas the energy system of GB differs from other countries (e.g. concerning the generation mix or dependency on an individual fuel type), similar questions surrounding flexibility, decentralisation and variability may be explored.

The linking of energy-transport models allows a comprehensive analysis of energy impacts of ambitious heat and transport decarbonisation strategies, which is typically lacking. Results from such an integrated approach provide evidence to develop coherent policies to meet the net-zero emissions target. Future energy systems are likely to be characterised by spatio-temporal interactions between different vectors through technologies such as CHP, hydrogen production via electrolysis and end-demand through electric vehicles. The simulation of linkages and reliance of technologies across multiple energy vectors demonstrates, for example, the production of hydrogen from renewable electricity can lead to a significant reduction in curtailments. Such results are not evident from individually modelled energy vectors. In addition, transmission and distribution system modelling allowed the exploration of centralised and decentralised operation of the entire energy system. Exploring an electrification and multi-vector energy strategy to meet heating and transport requirements in 2050 indicates that decentralised operation and distributed generation leads to a reduction in energy curtailments. This reduces the need for investment in network capacity as observed across both strategies. For the Electric Strategy, reduced generation from fossil-fuelled peak generation plants was simulated, thus reducing residual emissions. The Multi-vector Strategy leads to greater interactions between gas systems and hydrogen production and storage, as electricity generation is prioritised for local heating and transport demand. Decentralised operation also encouraged Energy hubs to supply energy to other regions through the transmission network for meeting demands.

One key advantage of performing integrated system analysis is the ability to explore vulnerabilities that might manifest in one energy system or vector and impact other systems. This was seen in the Multi-vector Strategy, where a lack of renewable generation in some regions leads to a shift from electrolysis to SMR in the production of hydrogen, increasing natural gas supplies, associated emissions, and operating costs. Understanding how the energy system interacts with the transport sector is crucial for decision-makers, especially with the expected rapid growth in electric vehicles. Modelling spatial and temporal variability of energy supply across scales is essential for understanding transmission and distribution network capacity bottlenecks and supporting the operation and planning of a net zero energy system. The ability to manage distinct transmission level and Energy Hub (distribution) assets such as power plants and storage systems allows exploration of variable tariffs on transmission to distribution electricity flows.

In summary, modelling a future energy system that account for the variability of wind, flexibility options and the implications of decentralised energy system operation requires the representation of multiple energy vectors across scales and integration of key sectors such as transport.

## Supplementary Information

Supplementary Information.

## Data Availability

The workflows, code and data used are available from the corresponding author upon reasonable request.
