# Supplementary material for: Simulating flexibility, variability and decentralisation with an integrated energy system model for Great Britain
Source: Sci Rep. 2023 Mar 23;13:4772. doi: 10.1038/s41598-023-31257-9 (PMC10036483; doi:10.1038/s41598-023-31257-9)
Supplement: Supplementary file 1 — Supplementary Information. [file 41598_2023_31257_MOESM1_ESM.docx]

Supplementary Information

**Simulating flexibility, variability and decentralisation with an integrated energy system model for Great Britain**

Modassar Chaudry^1*^, Lahiru Jayasuriya^1,4^, Jim W. Hall^2^, Nick Jenkins^1^, Nick Eyre^2^, Sven Eggimann^2,3^

^1^School of Engineering, Cardiff University, Queen's Buildings, The Parade, Cardiff CF24 3AA, Wales, UK.

^2^ Environmental Change Institute, University of Oxford, South Parks Road, Oxford OX1 3QY, United Kingdom.

^3^ Urban Energy Systems Laboratory, Swiss Federal Laboratories for Materials Science and Technology, Empa, Dübendorf, Switzerland.

^4^ Department of Electrical and Electronics Technology, Faculty of Technology, Rajarata University of Sri Lanka.

*Corresponding author: School of Engineering, Cardiff University, Queen's Buildings, The Parade, Cardiff CF24 3AA, Wales, UK. E-Mail: ChaudryM@cardiff.ac.uk

**Supplementary Note A: Energy supply model**

The energy supply model performs operation optimisation over a given time-horizon, which in this study is a year. The objective function as given in the methods section in the main document (Equation 1) is subject to constraints^1–3^ related to the operation of the combined gas and electricity transmission networks and Energy Hub as outlined below.

Constraints from operating the natural gas transmission system:

- Gas supply from reception terminals, gas storage facilities and linepack in the pipelines are equal to the gas demand for large industrial consumers, power generation and gas flows into the energy Hubs.
- The gas supplies into the reception terminals are subjected to the availability of gas resources such as imported Liquified Natural Gas (LNG), gas production from the United Kingdom Continental Shelve (UKCS) and pipeline imports from European Countries.
- The gas flow within pipes satisfies the gas flow equation and determines nodal pressures at both ends of a pipeline. The gas flow is kept within the maximum gas flow capacity of each pipeline. The nodal pressure levels are kept within the operating pressure limits.
- The operation of a gas compressor station is limited by the maximum and minimum power requirements of the prime movers, and the overall compression ratio.
- The stored gas volume is balanced with gas withdrawn and gas injected from each gas storage facility.

Constraints from operating the electricity transmission system:

- Electricity generation from generation plants and interconnector imports is equal to the electricity demand for industrial consumers, electricity flows into the Energy Hubs and exports.
- The power output from a generator is constrained by its rated capacity and for interconnectors, by the rated capacity of the interconnector network link.
- The electricity output from wind and PV generators is variable with respect to input wind speed and solar irradiance at each busbar.
- The power flow in each transmission line is kept within the maximum power transfer capacity.
- The thermal power generators (combined cycle and open cycle gas turbines, coal and oil) adhere to the physical limits of ramping up/down and minimum start-up/shutdown times to balance the intermittency of wind and PV power generation.
- A minimum reserve level is set for thermal generators for contingencies such as unplanned power supply outages and variations in power supply and demand.
- The stored electrical energy is balanced with dispatched power and pump power of each pumped storage facility.
- The total cooling water withdrawn for cooling thermal power stations is constrained by the maximum water availability for the electricity sector at each busbar.

Constraints from operating the Energy Hubs:

- Supply and demand are balanced for electrical, natural gas, heat and hydrogen energy within each Energy Hub.
- The electrical and gas energy supplies from the transmission networks are kept within the rated supply capacity of each gas offtake node and electricity busbar connected to the Energy Hub.
- The energy output from each technology type is kept within the rated output capacity.
- The electrical energy output from wind and PV plants is variable with respect to input wind speed and solar irradiance at each energy hub.
- Input and output energy conversion relationships are satisfied via energy efficiencies for each technology type.
- Heat and electrical energy outputs from combined heat and power technologies satisfy the heat to power ratio.
- Energy stored is balanced with input and output energy flows from each energy storage facility.
- The use of biomass and waste to energy technologies is constrained by the availability of biomass and solid waste fuels within the Energy Hub.

**Representation of the electricity and natural gas transmission networks and Energy Hub regions in Great Britain.**

The electricity transmission network is represented by a network with 29 bus bars and approximately 50 transmission lines^4^. The network represents data from actual 275kV/400kV grid substation locations across the current electricity transmission network^5,6^ and power stations^7^.

The natural gas transmission network is represented by a network with 80 nodes and 110 pipes. The representative simplified network was developed by using the network data and maps published by the gas network operator^8^. This simplified representation was adopted from the previous literature^9,10^. The representative network preserves the actual locations of gas offtake nodes, storage facilities, gas terminals and compressor stations in GB.

An Energy Hub provides an aggregated view of energy supply and demand within local energy systems within a specified geographic region. The energy supply represents an aggregated installed capacity of electricity generation technologies, heat supply technologies, gas storage, and the total capacity of electricity and natural gas transmission network supply points. Energy demands are aggregated for heating (space heating and hot water) and non-heating (consumer electronics, cooking, electric vehicle charging, hydrogen vehicle refuelling) end-uses. Within Energy Hubs the physical networks such as electricity distribution, gas distribution, and heat networks are not modelled. Figure A.1 shows the chosen 29 different geographic regions^11^ to allow a 1 to 1 mapping of electricity transmission bus bars to the energy hub regions. Each energy hub region consists of different LADs to match the availability of census data, energy demand model data outputs, transport model outputs, and distributed generation capacity data^12,13^.


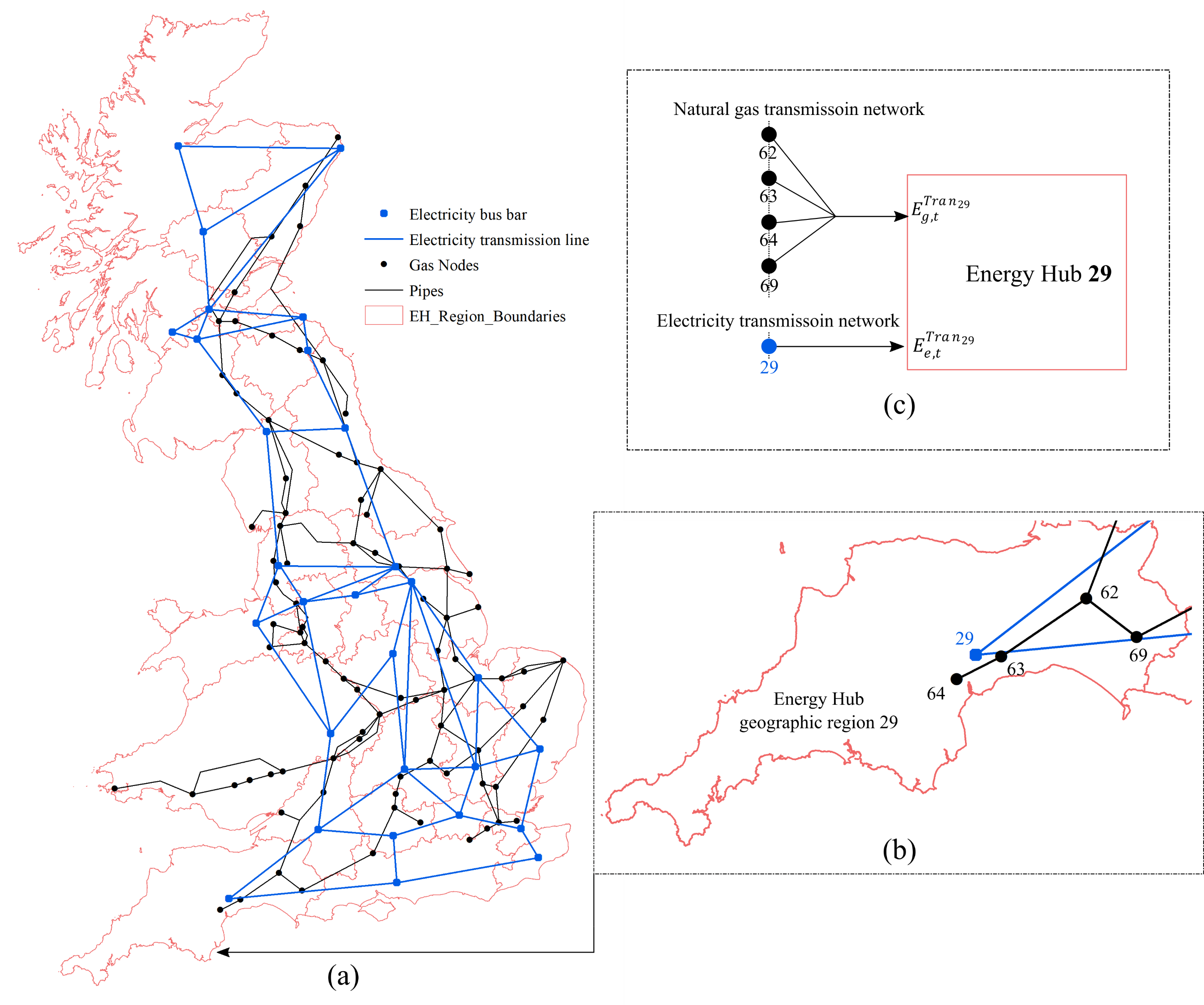


**Fig. A.1:** (a) Spatial map of energy hub regions, and gas and electricity transmission networks, (b) example spatial map of the Energy Hub 29 with the transmission networks, and (c) representation of energy flow from transmission networks to Energy Hub.

The layout of the Energy Hub used is shown in Figure A.2. This is a generic layout, and the actual layout changes with the available energy supply sources, technologies, storage systems and energy demand.


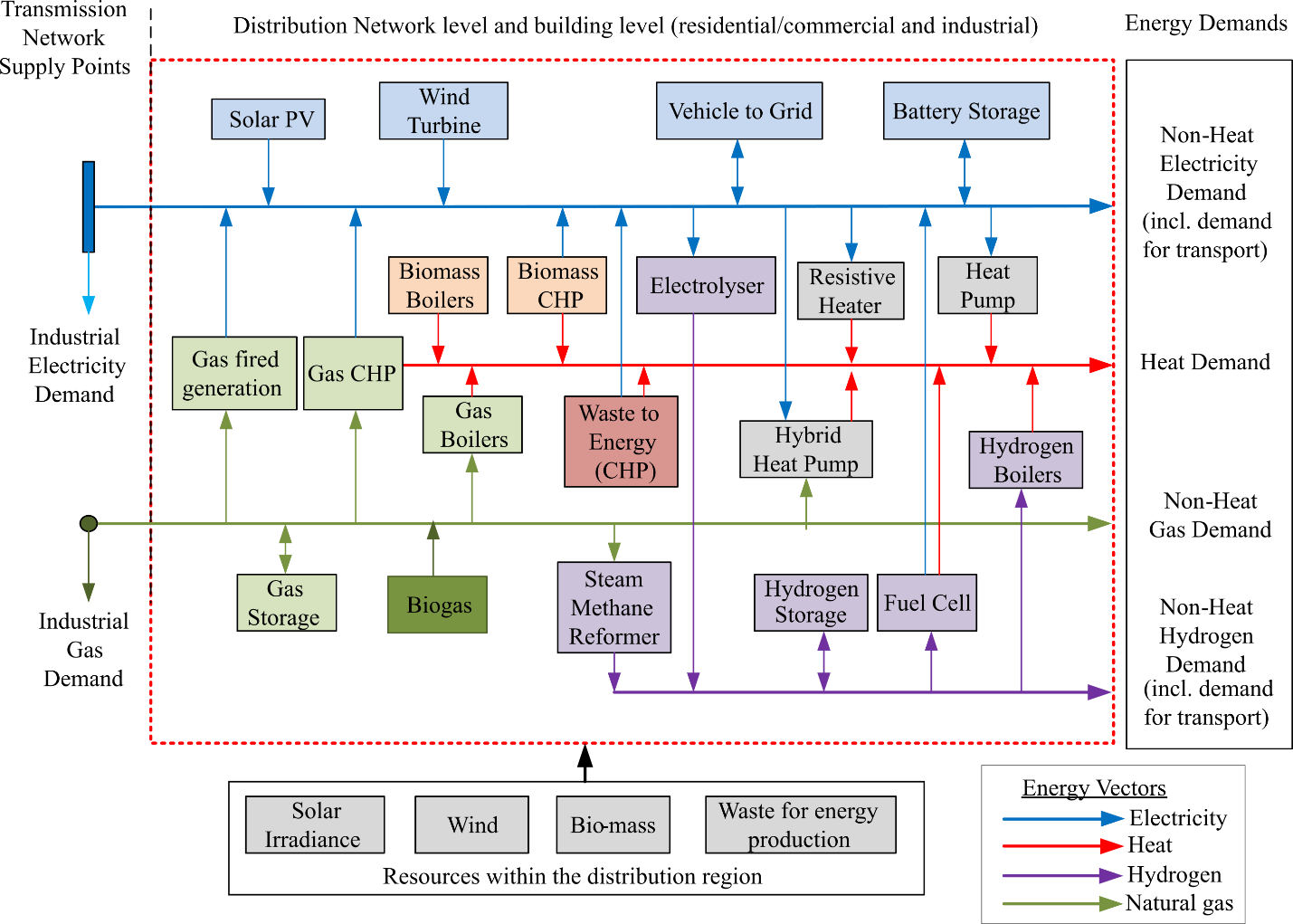


**Fig. A.2:** Energy Hub layout used in this study to model integrated local electricity, natural gas, heat, and hydrogen supply.

**Modelling of hydrogen production methods**

Production of hydrogen using electrolysis and steam methane reformation (SMR) is modelled by considering hydrogen transportation through distribution systems in the Energy Hubs such that supply meets heating, non-heating and transport hydrogen demands.

**Modelling of storage facilities**

Storage facilities for natural gas, electricity (in grid-scale batteries), and hydrogen supply systems are modelled in the energy supply model. Both short-term (intraday) and seasonal natural gas storage operations are represented. The operation of grid-scale battery storage systems is modelled including their operation to store excess renewable generation during off-peak hours and withdraw power during peak hours. Hydrogen storage facilities are considered, allowing storage of hydrogen produced via electrolysis using excess renewable electricity and through SMR during mainly off-peak hours. Seasonal gas storage facilities are connected to natural gas transmission networks. Both grid-scale electric batteries and hydrogen storage facilities are connected locally within the Energy Hubs. Electric vehicle charging and hydrogen re-fuelling demands are represented within the energy hubs

**Supplementary Note B: Energy supply strategies**

Tables B.1 – B.6 provide detailed information on the parametrisation of the Electric and Multi-vector Strategy for all used models, i.e., the transport model (Table B.1), the energy demand model (Table B.2) and the energy supply model (Tables B.3 – B.6).

**Table B.1** – **Parameterisation of the transport model:** Fraction of vehicles by engine types for each energy supply strategy in 2050.

| **Vehicles by engine type** | **Unit** | **Multi-vector Strategy** | **Electric Strategy** |
| --- | --- | --- | --- |
| **Car** |  |  |  |
| Battery Electric Vehicles | % | 50 | 100 |
| Plug-in hybrid electric vehicles | % | 14 | 0 |
| Fuel Cell electric vehicles | % | 35 | 0 |
| Hybrid electric vehicles and LPG | % | 1 | 0 |
| Internal Combustion Engines | % | 0 | 0 |
|  |  |  |  |
| **Van** |  |  |  |
| Battery Electric Vehicle | % | 55 | 100 |
| Internal Combustion Engines-diesel | % | 0 | 0 |
| Plug-in hybrid electric vehicle - diesel | % | 0 | 0 |
| Fuel Cell electric vehicle | % | 45 | 0 |
|  |  |  |  |
| **Heavy Goods Vehicles** |  |  |  |
| Internal Combustion Engines - diesel | % | 0 | 5 |
| Plug-in hybrid electric vehicle - diesel | % | 50 | 55 |
| Fuel cell electric vehicle | % | 50 | 40 |

| **Model Variable** | | **Units** | **Multi-vector** | **Electric** |
| --- | --- | --- | --- | --- |
| Residential | Smart meter roll-out | % | 100 | |
|  | End use specific reduction | % | 25 | |
|  | Achieved technological efficiency | % | 40 | |
|  | Improved insulation | % | 40 | |
|  | Improved thermal comfort | C | 1 | |
|  | Switching to LED | % | 100 | |
|  | | | | |
| Service | Smart meter roll-out | % | 100 | |
|  | End use specific reduction | % | 40 | |
|  | Achieved technological efficiency | % | 20 | |
|  | Improved Insulation | % | 40 | |
|  | Improved thermal comfort | C | 1 | |
|  | Switching to LED | % | 100 | |
|  | | | | |
| Industry | Achieved technological efficiency | % | 40 | |
|  | End use specific reduction | % | 20 | |
|  | Improved Insulation | % | 40 | |
|  | Improved thermal comfort | C | 1 | |
|  | Green gas (bio-energy) substitution of gas and solid fuel | % | 0 | |
|  | Hydrogen substitution of gas and solid fuel | % | 35 | 0 |

**Table B.2** – **Parametrisation of the energy demand model**: Parameters used to calculate final energy demand in 2050 for each energy supply strategy

The following assumptions/considerations were made for the different model variables listed above.

- Smart meter rollout: all-end uses will have a 3% energy saving with the roll-out of a smart meter.
- End use specific reduction: These include the energy savings from consumer electronics and computing.
- Achieved technological efficiency: Energy savings through technology and efficiency improvements are modelled only for selected end uses. In the residential sector, it is for lighting, cooking, space and water heating. In the commercial/services sector space and water heating, cooling and humidification are considered. Space heating is considered for the industrial sector.
- Improved insulation: modelled as a reduction in base temperature.
- Improved thermal comfort: Energy savings in residential, service and industrial space and water heating are considered.

| **Heating Technology** | **Base Year (GW)** | **Electric (GW)** | **Multi-vector (GW)** |
| --- | --- | --- | --- |
| ASHP + GSHP | 0.274 | 25.236 | 9.013 |
| Gas Boiler - Building | 43.951 | - | 24.267 |
| Electric boiler - Building | - | 4.056 | - |
| Resistive heating - Building | 5.494 | 3.605 | - |
| Hydrogen Boiler - building | - | - | 10.140 |
| Hybrid Heat Pump - building | - | 4.056 | - |
| Oil Boiler - Building | 6.498 | - | - |
| Gas CHP - DH | 0.030 | - | 2.028 |
| Biomass CHP - DH | 0.070 | - | 4.636 |
| Waste CHP - DH | - | - | 4.636 |
| Gas Boiler - DH | 0.031 | - | - |
| Heat Pump -DH | - | - | 4.056 |
| H2 Fuel Cell - DH | - | - | 6.953 |
| **Total Capacity** | **56.317** | **36.993** | **65.729** |

**Table B.3** – **Parameterisation of the energy supply model**: Heat supply capacity (MW_th_) for the base year and energy supply strategies in 2050.

**Table B.4** – **Parameterisation of the energy supply model:** National electricity supply capacity (MW) for the base year and energy supply strategies in 2050.

| **Generation Type** | **Base Year (MW)** | **Electric (MW)** | **Multi-vector (MW)** |
| --- | --- | --- | --- |
| Oil | 867 | 0 | |
| Gas CCS | 0 | 42900 | |
| Coal | 17284 | 0 | |
| Gas (CCGT + OCGT) | 26962 | 1000 | |
| Hydro | 1193 | 1297.3 | |
| Pumped Hydro | 2744 | 5813.9 | |
| Interconnectors | 3950 | 20055 | |
| Other (tidal and marine) | 0 | 3892 | |
| Nuclear | 8985 | 18600 | |
| Onshore wind | 4156 | 17173.9 | |
| Offshore wind | 4335 | 61982.4 | |
| Solar | 0 | 892.8 | |
| Battery | 0 | 5271.6 | |
| BECCS | 0 | 7000 | |
|  | | | |
| Total Transmission | *70,476* | *185,879* | |
|  | | | |
| Gas (non-CHP) | 1341.1 | 1500 | 1500 |
| Onshore Wind | 3714.4 | 11401.2 | 11401.2 |
| Offshore Wind | 536.4 | 942.4 | 942.4 |
| PV | 6734.8 | 41122.9 | 41122.9 |
| CHP gas | 2294.1 | 0 | 0 |
| Oil (Diesel etc.) | 437.9 | 0 | 0 |
| Biomass other | 413.9 | 1307.5 | 1307.5 |
| Biomass CHP | 0 | 977.2 | 3090 |
| Waste Other | 0 | 2373.3 | 2373.3 |
| Waste CHP | 748.4 | 934.2 | 3090 |
| Fuel Cells | 0 | 1.3 | 4635 |
| Vehicle to Grid | 0 | 9940.4 | 9940.4 |
| Storage (battery) | 0 | 11924.3 | 11924.3 |
| Other | 41 | 101.8 | 101.8 |
| Total distribution (GW) | *16,262* | *82,525* | *91,429* |
|  |  |  |  |
| **Total capacity** | **86,738** | **268,405** | **277,308** |

**Table B.5** – **Parameterisation of the energy supply model:** Natural gas supply by source for the base year and energy supply strategies in 2050

| **Natural Gas Source** | **Base Year**  **(bcm/year)** | **Electric / Multi-vector in 2050**  **(bcm/year)** |
| --- | --- | --- |
| UKCS | 36.1 | 1.4 |
| Shale | 0.0 | 0.00 |
| Green Gas (Biogas) | 0.0 | 6.7 |
| Imports (Pipeline and LNG) | 45.4 | 55.2 |
| **Total** | ***81.5*** | ***63.3*** |

**Table B.6** – **Parameterisation of the energy supply model:** Hydrogen production capacity by technology in 2050

| **Hydrogen Production Technology** | **Electric (GW)** | **Multi-vector (GW)** |
| --- | --- | --- |
| Steam Methane Reformation | 1 | 25 |
| Electrolysis | 2 | 60 |
| **Total** | ***3*** | ***85*** |

The following socio-economic parameters (Table B.7) are defined as model inputs for all three models:

**Table B.7 – Socioeconomic parameters**

| **Model variable** | **Unit** | **Multi-vector – 2050** | **Electric – 2050** |
| --- | --- | --- | --- |
| Population | Million people | 66.4 | 96.6 |
| Gross Value Added (GVA) | £ person^-1^ | 37,035 | 30,338 |
| Oil price | $ bbl^-1^ | 60 | 85 |
| Coal price | $ tonne^-1^ | 66 | 87 |
| Gas price | pence therm^-1^ | 36 | 63 |
| Cost of carbon | £ tCO_2_e^-1^ | 18.31 | 39.41 |

Population and GVA data were adapted from Hall et. al (2016) ^14^ where Multi-vector Strategy uses population scenario F and the Electric Strategy uses population scenario D. The UK’s Department of Business, Energy and Industrial Strategy (BEIS) data projections were used for fossil fuel prices and carbon prices^15^ from which the low-scenario is used for the Multi-vector Strategy and central scenario is used for Electric Strategy.

**Supplementary Note C: Energy Demand and Transport Models**

**Energy demand model**

An existing spatial-temporally highly resolved energy demand simulation model^16^ is used to project future energy demand based on different socio-technical scenario assumptions concerning population, gross value added (GVA), technological efficiencies, changes in the technological mix per end-use consumption or behavioural change. Energy demands for each simulation year are projected relative to initial base year conditions in 2015. The model is based on a decomposition approach, distinguishing between residential, service and industry energy demands according to the Department for Business, Energy and Industrial Strategy (BEIS) for a total of 28 end uses 34 sectors and 7 fuel vectors^17^. For selected end uses such as heating, different technologies are configured. The model is a mixture of a top-down and bottom-up model, as end use demands are derived from national energy demand consumption statistics and specific load profile data per technology or end-use that are provided at a disaggregate level^18^.

A three-step process is utilised to obtain hourly and regional energy demand data. Firstly, national energy demand statistics are disaggregated into 391 local authority districts (LAD) based on disaggregation factors. Secondly, future demand is projected relative to base year demands in a back-casting approach, where the uptake for each simulation year is based on scenario drivers according to changes in the dwelling stock, temperatures, technological efficiencies, the technology mix or behavioural change. Thirdly, regional annual demand data are disaggregated to hourly temporal resolution based on end-use and technology-specific load profiles, which are collected from different measurement trial data. For space and water heating, heating degree day calculations are used for the disaggregation of annual to daily demand. Sub-national non-residential gas and electricity demand^19^ is used for calibration to improve the spatial disaggregation of non-residential energy demands. The model is fully described in Eggimann et al.^16^.

**Transport model**

The model^20^ used is the strategic road transport model for Great Britain produced by the Infrastructure Transitions Research Consortium (version 2). This road network model covers all major roads in Great Britain, with this network being superimposed on a zoning structure based on LADs. A base year origin-destination road trip matrix was generated, allocated to the road network, and calibrated against traffic count data to provide a representation of initial traffic levels on all network links. The model allows for simulating changes in traffic levels and transport energy consumption in response to changes in population, economic activity, travel time and costs using an elasticity-based approach. This involves calculating new traffic levels for each flow in the origin-destination matrix using Equation C.1 which are then reassigned to the network using a probabilistic process based on the relative attractiveness of different route options in terms of time and cost.

| $F_{ijy}=F_{ijy-1}\left( \frac{P_{iy}+P_{jy}}{P_{iy-1}+P_{jy-1}} \right)^{\eta_{P}}\left( \frac{I_{iy}+I_{jy}}{I_{iy-1}+I_{jy-1}} \right)^{\eta_{I}}\left( \frac{T_{ijy}}{T_{ijy-1}} \right)^{\eta_{T}}\left( \frac{C_{ijy}}{C_{ijy-1}} \right)^{\eta_{C}}$ | (C.1) |
| --- | --- |

Where $F_{ijy}$is the flow between origin zone *i* and destination zone *j* in year *y*; $P_{iy}$ is the population in zone *i* in year *y*; $I_{iy}$ is the GVA per head in zone *i* in year *y*; $T_{ijy}$ is the average travel time between zone *i* and zone *j*; $C_{ijy}$ is the average travel cost between zone *i* and zone *j*; *η* is a demand elasticity.

The assignment of flows to the network leads to changes in travel time and cost on individual network links as a result of changing congestion levels. The relative costs of different route options for each flow are recalculated based on updated times and costs, and new flow times and costs are fed back into Equation C.1 to further alter traffic levels. The model simulates the energy consumed for each trip, covering all road vehicle power sources, including electricity and hydrogen.

In this study several ‘scenario’ variables were prespecified at the start of the model run, including changes in the relative fuel efficiency of vehicles over time, and the proportion of vehicles powered by different fuels in each future year (e.g., 50% battery-electric, 30% hybrids and 20% internal combustion). This allows investigation of the impacts of changes in vehicle fuel efficiency and power source on outputs such as energy consumption and carbon emissions. The outputs can be disaggregated to various levels of spatial detail, with energy consumption reported at the LAD level.

**Energy demand for transport and vehicle to grid modelling and assumptions**

For this study, the input parameters were defined according to the two Strategies given in Table B.1. Using these inputs, the transport model provides the number of vehicle trips (disaggregated by engine type, i.e. electric, hydrogen, internal combustion engines) and energy consumed (electricity and hydrogen) for each trip within each LAD during each hour across weekdays and weekends during a year.

These outputs are then translated into electricity and hydrogen demand for transport, and the availability of EV batteries for V2G services using an energy-transport module. The energy-transport module uses the following assumptions.

- A trip-to-vehicle ratio of one and a high probability that most trips are local
- An average electric car battery capacity of 30kWh^21^
- About 20% of stationary vehicles provide Vehicle to Grid at a power output of 7kW^22^

The energy consumed in vehicles (electrical and hydrogen) was first translated to a daily energy demand for transport by summing across 24 hours. To generate unmanaged hourly transport energy demands, normalised profiles^23^ as shown in Figure C.1 were used. The profiles are normalised by the total daily energy demand and consider the difference between weekdays and weekends. These profiles are superimposed with the calculated total daily energy demand for transport to produce hourly unmanaged electricity and hydrogen demands for transport, which is then provided as input to the energy supply model.

Fig. C.1: Normalised hourly electric vehicle charging, and hydrogen vehicle re-fuelling demand profiles for weekdays and weekends used in the study


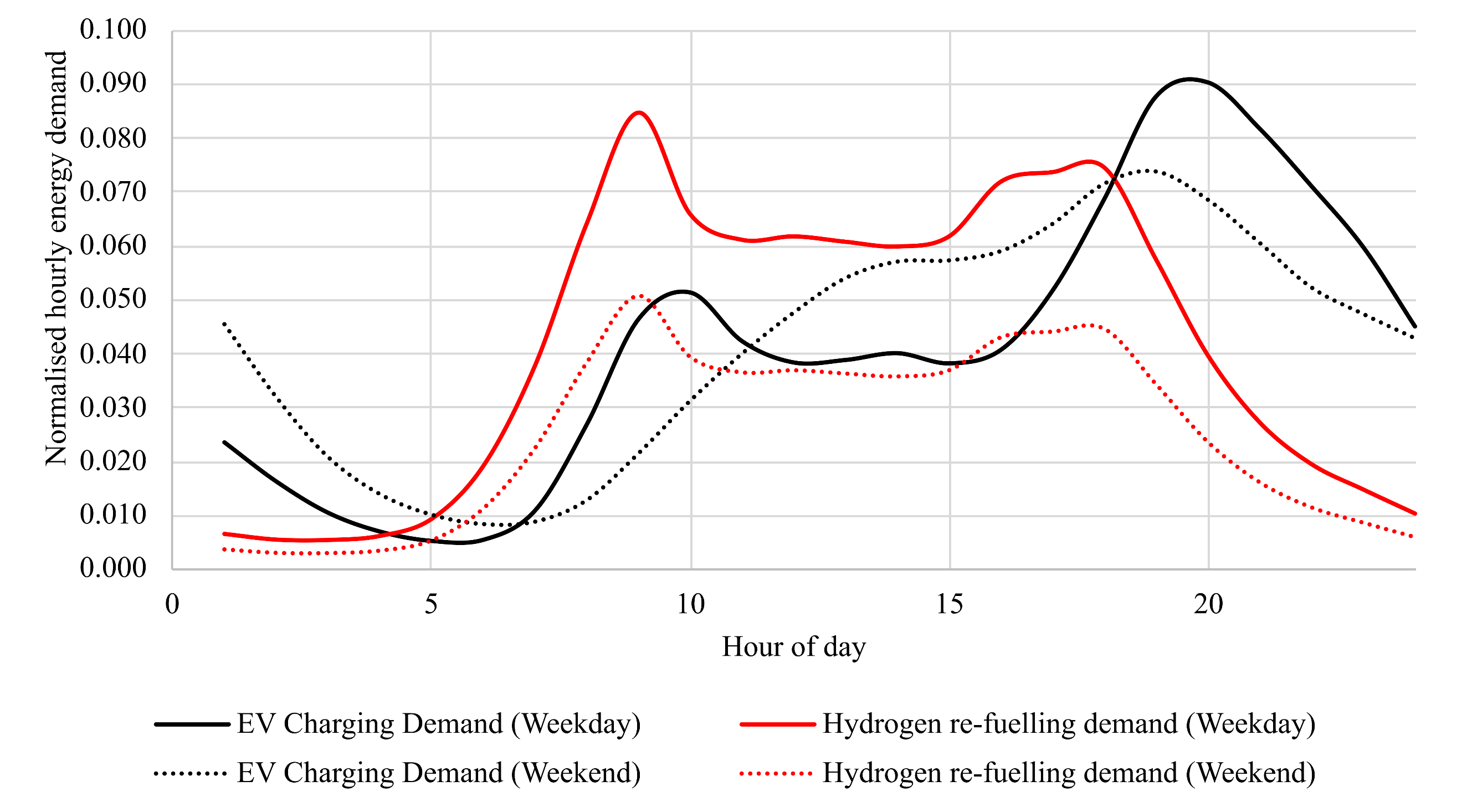


Modelling of managed EV charging does not use a fixed profile, as described in the unmanaged charging case. Here, a decision variable is defined for the EV charging demand, which is summed over 24 hours and equals the daily EV charging demand from the transport model. Managed charging assumes that EVs charge when there is plentiful renewable electricity available, for example during off-peak periods and when electricity generation costs are low.

The energy-transport module calculates the availability of electrical energy in the EV batteries for V2G services, and its variability during the day with respect to daily travelling behaviour. The electrical energy used for EV trips (red bars) and the electrical energy available to provide V2G services (black bars) is shown in Figure C.2.

Fig. C.2: Illustration of electrical energy available in EV batteries for vehicle to grid


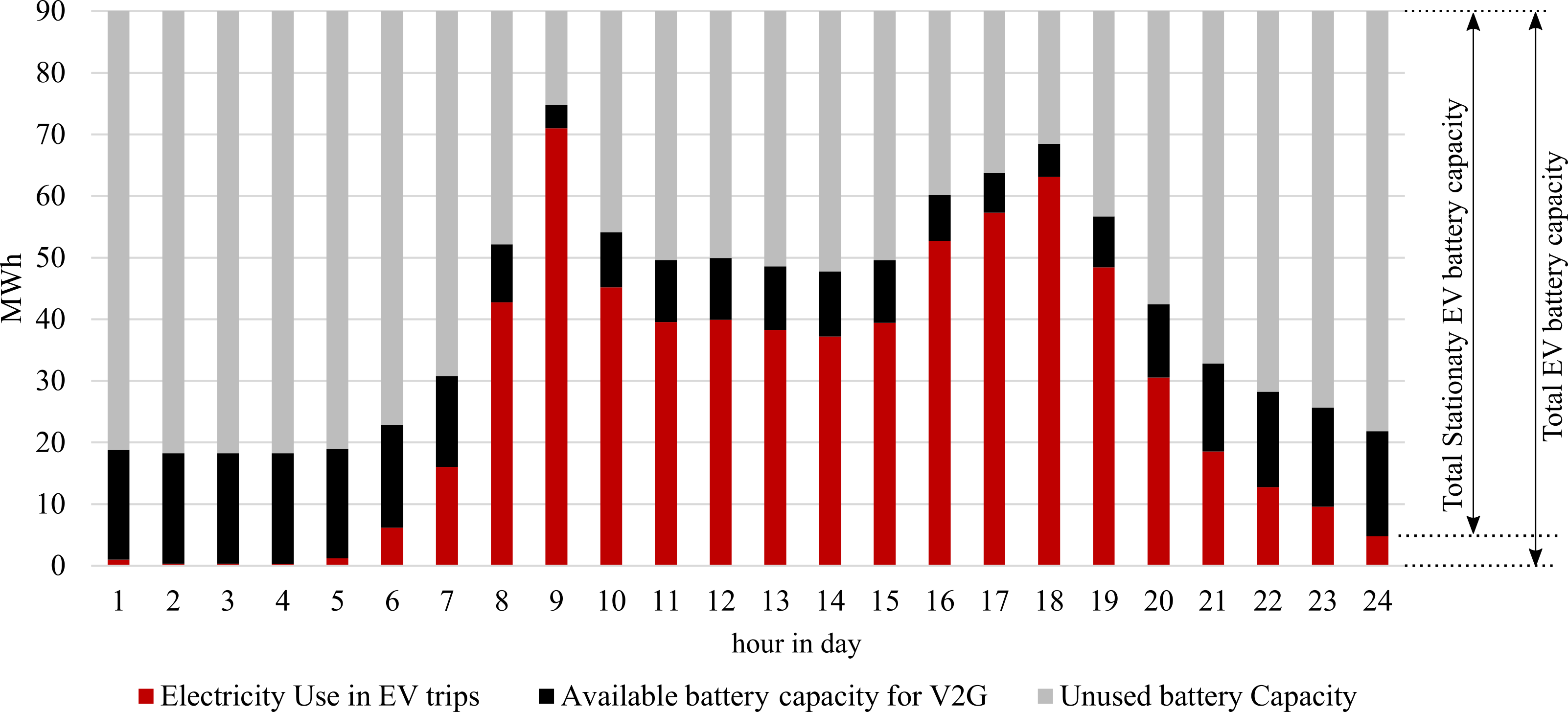


**Supplementary Note D: Additional Results / Tables**

Table D.1 shows a summary of the key modelling metrics to compare the impact of centralised /decentralised operation of the two distinct energy supply strategies on the GB energy system in 2050.

**Table D.1 –** A summary of key modelling metrics comparing centralised and decentralised operations of the GB energy system for the two strategies in 2050. Change in decentralised operation compared to the centralised operation is presented in brackets either as an increase (green) or decrease (red).

| **Metric (Annual)** | **Electric strategy** | **Multi-Vector strategy** |
| --- | --- | --- |
| Primary energy supply (TWh) | 797.4 (-2.4) | 1028.9 (5.7) |
| Transmission system electricity generation (TWh) | 389.4 (-20.5) | 448.9 (-25.1) |
| Distribution system electricity generation (TWh) | 158.3 (23.5) | 133.6 (3.8) |
| Natural gas fired peaking plant electricity generation (TWh) | 17.2 (-2.3) | 0 (N/A) |
| Renewable electricity curtailed (TWh) | 56.5 (-5.3) | 13.1 (-2.9) |
| Electrical energy from Batteries (TWh) | 8.8 (4.5) | 3.5 (0.6) |
| Natural gas supply (TWh) | 125.5 (-3.1) | 190.3 (21.2) |
| Hydrogen supplied from storage facilities (TWh) | 2.2 (1.2) | 70.6 (16.9) |
| Emissions (ktCO_2_) | 17,910 (-30) | 25,486 (195) |
| Cost of system operation (M£) | 22,008 (-194) | 31,459 (580) |

**Supplementary Note E: Wind power error analysis**

Wind Turbine details ^24^ used are given below.

| Wind turbine rated capacity, $P_{k}^{rated}$ | : 2MW |
| --- | --- |
| Rated wind speed, $v^{rated}$ | : 11m/s |
| Cut-in wind speed, $v^{cut-in}$ | : 4m/s |
| Cut-off wind speed, $v^{cut-off}$ | : 20m/s |
| The swept area of the rotor, $A_{k}$ | : 6362m^2^ |

Air density $\rho$ is taken as 1.225kg/m^3^, and the power coefficient $C_{p}$ is taken as 0.45. When wind speed $v_{t}$ is $v^{cut-in}\leq v_{t}\leq v^{rated}$, the power output from the wind turbine $P_{k,t}$is calculated from^25^ (Equation E.1),

| $P_{k,t}=C_{p}\times\frac{1}{2}\times\rho\times A_{k}\times v_{t}^{3}$ | E.1 |
| --- | --- |

And the linear approximation used in the model calculates the power output by (Equation E.2),

| $P_{k,t}=\left( \frac{v_{t}-v^{cut-in}}{v^{rated}-v^{cut-in}} \right)P_{k}^{rated}$ | E.2 |
| --- | --- |

Figure E.1 shows the comparison between actual and linearly approximated power output curves.


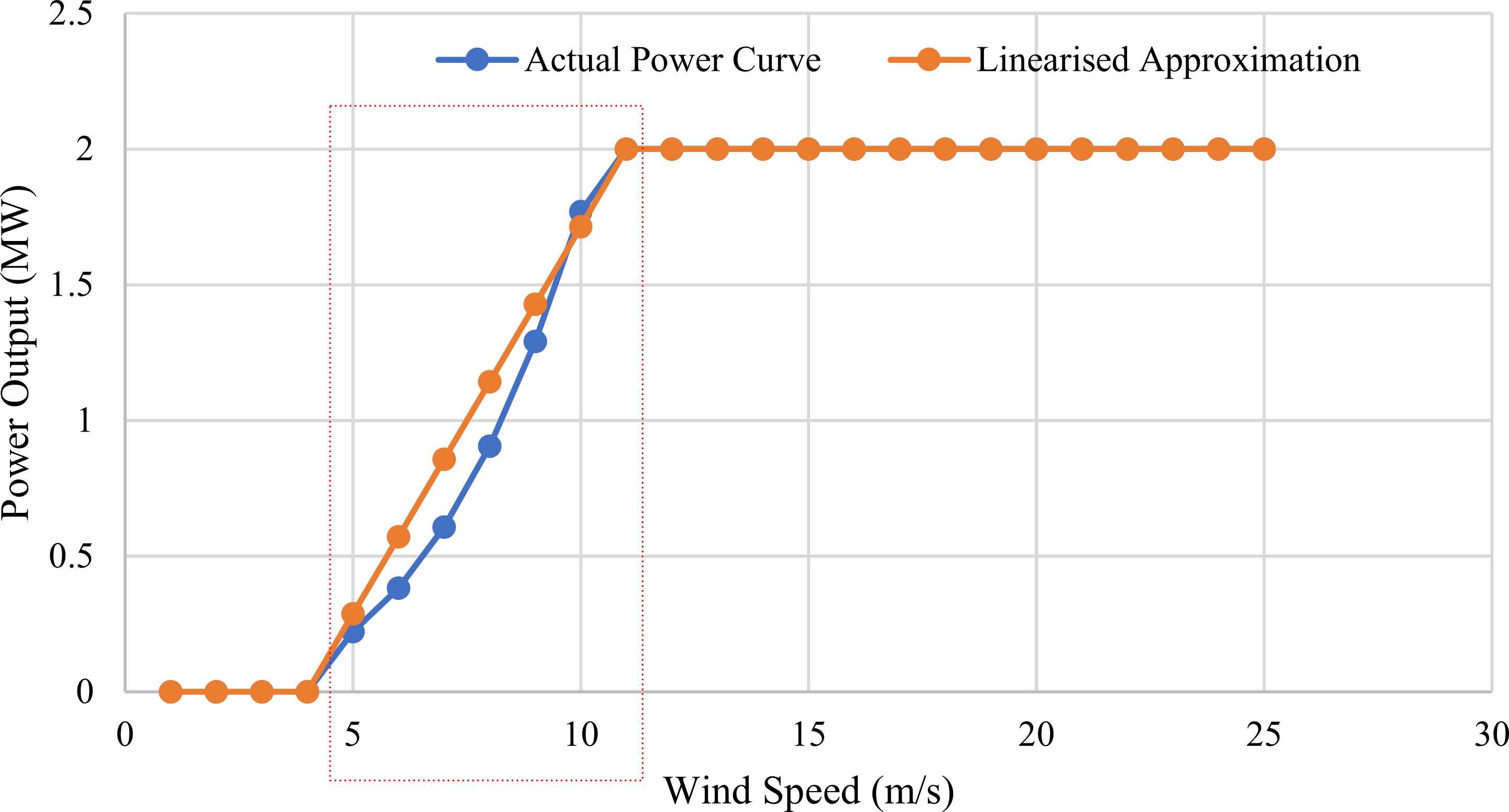


**Fig. E.1:** Wind power vs wind speed curve comparison between actual power curve (Given by Equation D.1) and the linear approximation (given by the Equation D.2) used in the model.

Table E.1 shows the values used to plot Figure E.1 in detail and quantifies the error due to the linear approximation.

Table E.1. Calculated error when wind speed $v_{t}$ is $v^{cut-in}\leq v_{t}\leq v^{rated}$ using the linear approximation.

| Wind Speed (m/s) | Actual power output (MW) given by Equation E.1 | Linearised approximation for power output (MW) given by Equation E.2 | Error % |
| --- | --- | --- | --- |
| 4 | 0 | 0 | 0 |
| 5 | 0.221 | 0.287 | 23.11 |
| 6 | 0.382 | 0.571 | 33.10 |
| 7 | 0.607 | 0.857 | 29.18 |
| 8 | 0.906 | 1.143 | 20.72 |
| 9 | 1.290 | 1.429 | 9.69 |
| 10 | 1.769 | 1.714 | 3.23 |
| 11 | 2 | 2 | 0 |

**Supplementary Note F: Data and model availability**

The three models: Energy Demand, Energy Supply and Transport Models are part of the National Infrastructure Systems Model (NISMOD).

The model codes, data and their installation instructions are available in the public NISMOD GitHub repository: <https://github.com/nismod/nismod2> and further documentation is available at <https://nismod.github.io/nismod2/>.

For each model, the documentation can be accessed as follows:

**Energy Demand** Model: <https://ed.readthedocs.io/en/latest/documentation.html> and <https://nismod.github.io/nismod2/energy-demand.html>

**Energy Supply** Model: <https://nismod.github.io/nismod2/energy-supply.html>

**Transport** Model: <https://nismod.github.io/nismod2/transport.html>

Simulation Model Integration Framework (**SMIF**): <https://smif.readthedocs.io/en/latest/>

**References**

1. Chaudry, M., Jenkins, N. & Strbac, G. Multi-time period combined gas and electricity network optimisation. *Electr. Power Syst. Res.* **78**, 1265–1279 (2008).

2. Qadrdan, M., Wu, J., Jenkins, N. & Ekanayake, J. Operating strategies for a gb integrated gas and electricity network considering the uncertainty in wind power forecasts. *IEEE Trans. Sustain. Energy* **5**, 128–138 (2014).

3. Jayasuriya, L. Multi-scale modelling of integrated energy supply systems. (Cardiff University, 2020).

4. Bell, K. R. W. & Tleis, A. N. D. Test system requirements for modelling future power systems. *IEEE PES Gen. Meet. PES 2010* 1–8 (2010). doi:10.1109/PES.2010.5589807

5. National Grid. National Grid Electricity Network route maps. (2017). Available at: https://www.nationalgridet.com/network-and-assets/network-route-maps. (Accessed: 1st February 2017)

6. National Grid. NETS ETYS 2015 - Appendix A - System Maps. 1–8 (2015).

7. Carbon Brief. UK Electricity Generation Map. (2016). Available at: https://www.carbonbrief.org/mapped-how-the-uk-generates-its-electricity.

8. National Grid. National Grid Gas Network route maps. (2018). Available at: https://www.nationalgridgas.com/land-and-assets/network-route-maps. (Accessed: 31st July 2018)

9. Qadrdan, M., Chaudry, M., Wu, J., Jenkins, N. & Ekanayake, J. Impact of a large penetration of wind generation on the GB gas network. *Energy Policy* **38**, 5684–5695 (2010).

10. Osiadacz, A. J. *Simulation and analysis of gas networks*. (Gulf Publishing Company,Houston, TX, 1987).

11. Jayasuriya, L. Multi-scale modelling of integrated energy supply systems. (Cardiff University, 2020).

12. BEIS. UK Energy in Brief. *UK Gov* 29–30 (2018).

13. Green Alliance & RegenSW. *Local Authority renewable energy league tables - National Survey Database*. (2016).

14. Hall, J. W., Tran, M., Hickford, A. J. & Nicholls, R. J. *The Future of National Infrastructure : a System-of-Systems Approach*. (Cambridge University Press, 2016).

15. BEIS. *Short-Term traded carbon values*. (2018).

16. Eggimann, S., Hall, J. W. & Eyre, N. A high-resolution spatio-temporal energy demand simulation to explore the potential of heating demand side management with large-scale heat pump diffusion. *Appl. Energy* **236**, 997–1010 (2019).

17. BEIS. Energy consumption in the UK. (2018). Available at: https://www.gov.uk/government/statistics/energy-consumption-in-the-uk. (Accessed: 3rd August 2019)

18. Bhattacharyya Govinda, S. C. & Timilsina, R. *Energy Demand Models for Policy Formulation A Comparative Study of Energy Demand Models*. (2009).

19. BEIS. SUB-NATIONAL ELECTRICITY AND GAS CONSUMPTION STATISTICS Regional, Local Authority, middle and lower layer super output area. (2016).

20. Lovrić, M., Blainey, S. & Preston, J. A conceptual design for a national transport model with cross-sectoral interdependencies. *Transp. Res. Procedia* **27**, 720–727 (2018).

21. Safari, M. Battery electric vehicles: Looking behind to move forward. *Energy Policy* **115**, 54–65 (2018).

22. Imperial College. *Accelerated electrification and the GB electricity system*. (2019).

23. National Grid. Future Energy Scenarios 2019 Data WorkBook. *Future Energy Scenarios* (2019). Available at: https://www.nationalgrideso.com/documents/169951-fes-data-workbook-v30. (Accessed: 12th December 2019)

24. Vestas. *Product fact sheet - Vestas wind systems, 2MW platform*. (2017).

25. Jenkins, N. & Ekanayake, J. *Renewable Energy Engineering*. (Cambridge University Press, 2017). doi:DOI: 10.1017/9781139236256
